# Regulation of biomass degradation by alternative σ factors in cellulolytic clostridia

**DOI:** 10.1038/s41598-018-29245-5

**Published:** 2018-07-23

**Authors:** Lizett Ortiz de Ora, Raphael Lamed, Ya-Jun Liu, Jian Xu, Qiu Cui, Yingang Feng, Yuval Shoham, Edward A. Bayer, Iván Muñoz-Gutiérrez

**Affiliations:** 1 Department of Molecular Microbiology and Biotechnology, Tel Aviv University, Tel Aviv, Israel; 2 CAS Key Laboratory of Biofuels and Shandong Provincial Key Laboratory of Energy Genetics, Qingdao Institute of Bioenergy and Bioprocess Technology, Chinese Academy of Sciences, Qingdao, Shandong China; 3 Department of Biotechnology and Food Engineering, Technion-IIT, Haifa, Israel; 4 Department of Biomolecular Sciences, The Weizmann Institute of Science, Rehovot, Israel; 5 Present Address: Outreach Research Training and Minority Science Programs, Francisco Ayala School of Biological Sciences, University of California, Irvine, California USA

## Abstract

Bacteria can adjust their genetic programs via alternative σ factors to face new environmental pressures. Here, we analyzed a unique set of paralogous alternative σ factors, termed σ^I^s, which fine-tune the regulation of one of the most intricate cellulolytic systems in nature, the bacterial cellulosome, that is involved in degradation of environmental polysaccharides. We combined bioinformatics with experiments to decipher the regulatory networks of five σ^I^s in *Clostridium thermocellum*, the epitome of cellulolytic microorganisms, and one σ^I^ in *Pseudobacteroides cellulosolvens* which produces the cellulosomal system with the greatest known complexity. Despite high homology between different σ^I^s, our data suggest limited cross-talk among them. Remarkably, the major cross-talk occurs within the main cellulosomal genes which harbor the same σ^I^-dependent promoter elements, suggesting a promoter-based mechanism to guarantee the expression of relevant genes. Our findings provide insights into the mechanisms used by σ^I^s to differentiate among their corresponding regulons, representing a comprehensive overview of the regulation of the cellulosome to date. Finally, we show the advantage of using a heterologous host system for analysis of multiple σ^I^s, since information generated by their analysis in their natural host can be misinterpreted owing to a cascade of interactions among the different σ^I^s.

## Introduction

Bacteria can sense the extracellular environment and transmit information intracellularly by using different types of signal transduction mechanisms^1^. After sensing the environment, one type of response is regulation of genes at the level of transcription initiation by alternative sigma (σ) factors allowing bacteria to adjust their transcriptional programs to environmental changes^2,3^. σ factors are the key component of RNA polymerase, since they provide promoter specificity. All bacteria harbor one primary σ factor (known as the housekeeping σ factor, σ^70^ or σ^A^) that is responsible for basal expression level of most genes. When the environmental conditions change, the housekeeping σ factor is substituted by the alternative σ factors, thereby redirecting the RNA polymerase to alternative promoters of genes that will help the bacterium deal with the new environmental conditions^4^.

Cellulolytic clostridia are anaerobic bacteria that use plant cell-wall polysaccharides as carbon sources. The structural complexity and composition of their substrate, together with the anoxic conditions of their ecosystem, have generated selective pressures for the evolution of extracellular multi-enzyme nanomachines called cellulosomes for efficient degradation of plant cell-wall polysaccharides^5^. During the process of cellulosic biomass breakdown, cellulolytic clostridia control the type(s) of enzymatic subunits present in the cellulosome complex to suit the type(s) of polysaccharide(s) that are exposed during the degradation process^6,7^. In the cellulosome-producing bacteria *Clostridium* (*Ruminiclostridium*) *thermocellum*, the enzymatic composition of the cellulosome is probably regulated by a group of at least 6 paralogous alternative σ^I^ factors that are related to the *Bacillus subtilis* σ^I^ factor^8–12^. However, the regulons of this set of σ^I^s are poorly understood.

The features of the σ^I^s are consistent with almost all of the characteristics of the ECF (**e**xtra**c**ytoplasmic **f**unction) σ factors^2^. Both σ^I^s and ECF σ factors share the following features. (i) They usually autoregulate their own expression. (ii) They are usually located in an operon with an anti-σ factor gene. In the case of σ^I^ factors, the latter gene is termed *rsgI* (i.e., regulation of *sigI*) that controls the activity of its cognate σ factor. (iii) The anti-σ factor is composed of three parts: an extracytoplasmic sensory module(s), a transmembrane domain, and an intracellular domain that sequesters the σ factor. (iv) The σ factor is activated by inhibiting the activity of the anti-σ factor.

The main difference between σ^I^ and ECF σ factors resides in their protein structure. Alternative σ^I^ factors harbor only the σ_2_ domain of the σ^70^ family that is involved in the recognition of the −10 promoter element, and the σ_4_ domain is substituted by a C-terminal domain termed σ_I-C_ that is likely involved in the recognition of the −35 promoter element^8^. Taken together, the above-mentioned characteristics render σ^I^ factors unique members of the σ^70^ family.

Some cellulosome-producing clostridia are characterized by multiple σ^I^ factors. The σ^I^ factors of *C. thermocellum* are highly homologous with identities between 36 and 45% among them. This observation raises the question of how a set of highly homologous alternative σ^I^ factors can avoid crosstalk among each other. This is of particular interest in a bacterium like *C. thermocellum*, which contains 8 paralogous σ^I^s, as well as other cellulosome-producing clostridia [notably, *Clostridium clariflavum, Clostridium straminosolvens, Clostridium* sp. Bc-iso-3, *Acetivibrio cellulolyticus*, and *Bacteroides (Pseudobacteroides) cellulosolvens*] which also produce between 8 and 16 highly homologous alternative σ^I^ factors that are presumably involved in similar regulatory networks. Moreover, there are reports where a set of ECF σ factors exhibits high crosstalk^13–15^, wherein the latter set shows less conservation compared to that of the *C. thermocellum* σ^I^ factors. For example, the *B. subtilis* ECF σ^M^, σ^W^ and σ^X^ factors share identities of only 25 to 32% but present high regulatory overlap^14^.

It should be noted that there is continued controversy regarding the classification of the cellulosome-producing clostridia^16^, and, in particular, for those species that produce complex or multiplex cellulosomes, characterized by a multiplicity of scaffoldin genes^17,18^. In this context, *Acetivibrio cellulolyticus* and *Bacteroides cellulosolvens* were clearly misclassified in the original works^19,20^, and both species were later determined to be members of the greater clostridial assemblage^21^. These latter discrepancies were not fully resolved by the recent attempt to reclassify of *Bacteroides cellulosolvens* as *Pseudoacteroides cellulosolvens*^22^.

In the present work, we demonstrate how a collection of five alternative σ^I^ factors in *C. thermocellum*, namely σ^I1^, σ^I2^, σ^I3^, σ^I4^ and σ^I6^, regulate the expression of 17 genes encoding different cellulosomal components. This analysis shows for the first time a sophisticated regulatory network of several alternative σ factors, which control the enzymatic composition of the cellulosome. Furthermore, our results show that σ^I^ factors from cellulosome-producing bacteria use highly conserved promoter sequences to delimit the genes that are under control of a given σ^I^. Our findings indicate that the −35 promoter element, proposed to be recognized by the novel domain σ_I-C_, is critical for the specificity of each σ^I^ factor. This promoter element can be divided into two regions: a highly-conserved homopolymeric A-tract motif in the 3′ region, that we herein propose as a general motif for σ^I^-dependent promoter recognition, and a more divergent region upstream of the A-tract motif that provides specificity to each σ^I^ factor. By using this information, we identified the regulons of one σ^I^ factor in *P. cellulosolvens*, a bacterium that produces the most complex cellulosomal system described until now^23^. Our results provide a better view into the mechanisms used by multiple alternative σ^I^s to differentiate their corresponding regulons. This information is crucial for future efforts to predict regulons of multiple σ^I^ factors in cellulolytic clostridia.

## Results

### Deciphering the regulatory networks of σ^I^ factors

**Figure 1:**
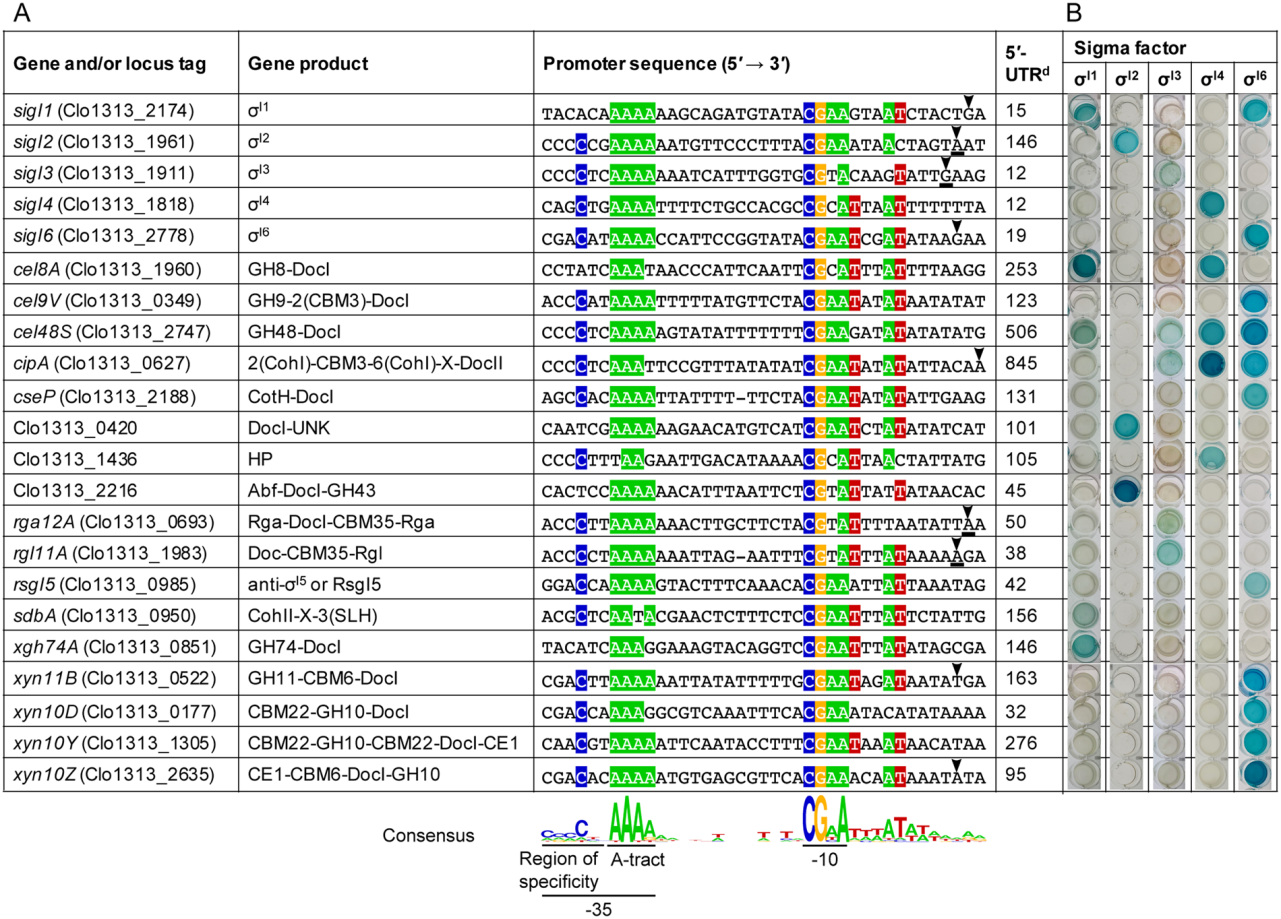
Regulons of *C. thermocellum* alternative σ^I^ factors. (**A**) Alignment of σ^I^-dependent promoters that were experimentally confirmed. The most conserved nucleotides are highlighted in color. TSSs are indicated by arrowheads and the TSSs identified in the present work (Supplementary Fig. S1) are underlined. The TSS of *sigI1*, *sigI6*, *cipA*, *xyn11B* and *xyn10Z* were identified in previous works^9,11,43^. Distances between the promoter sequences and the first codon of corresponding genes are shown in the column labeled 5′-UTR (5′-untranslated region). GH, glycoside hydrolase; Doc, dockerin; CBM, carbohydrate-binding module; Coh, cohesin; X, X-module (module of unknown function); CotH, spore coat protein H; UNK, unknown sequence; HP, hypothetical protein; Abf, Alpha-L-arabinofuranosidase; Rga, rhamnogalacturan acetylesterase; Rgl, rhamnogalacturonan lyase; SLH, S-layer homology domain; CE, carbohydrate esterase. (**B**) Recognition of σ^I^-dependent promoters by *C. thermocellum* σ^I^ factors. The respective promoters were fused to a LacZ reporter gene and their recognition by the different *C. thermocellum* σ^I^ factors was tested in a *B. subtilis* heterologous host, grown in 24-well cell culture plates with Spizizen’s minimal medium and X-gal. The development of blue color indicated the activation of the σ^I^-dependent promoters by a given *C. thermocellum* σ^I^ factor.

In a previous work, we predicted 40 putative σ^I^-dependent promoters in *C. thermocellum* by bioinformatic analysis^10^. To overcome the lack of convenient genetic tools to work directly in *C. thermocellum*^24^, we analyzed the recognition of the predicted promoters by *C. thermocellum* σ^I3^ and σ^I6^ in a heterologous *B. subtilis* host system^10^. This analysis revealed that the main types of enzymatic genes regulated by *C. thermocellum* σ^I3^ and σ^I6^ are pectin-degrading enzymes and xylanases, respectively^10^. In order to confirm the predicted σ^I^-dependent promoters of the spectrum of *C. thermocellum* σ^I^ factors that are proposed to be involved in the regulation of genes coding saccharolytic enzymes (σ^I1^ to σ^I6^)^9,10^, we herein mapped the transcriptional start sites (TSSs) of the *sigI2-rsgI2*, *sigI3-rsgI3*, *sigI4-rsgI4* and *sigI5-rsgI5* operons by the rapid amplification of cDNA ends (5′-RACE) technique (Supplementary Fig. S1). The TSSs of the *sigI1-rsgI1* and *sigI6-rsgI6* operons were mapped in a previous work^9^. During this analysis, however, we failed to identify a TSS of the *sigI4-rsgI4* and *sigI5*-*rsgI5* operons using this technique. The σ^I^-dependent promoter sequences associated with the TSSs mapped in the present work are shown in Fig. 1A.

Subsequently, in order to decipher the regulatory networks of *C. thermocellum* σ^I^ factors, we tested the recognition of the previously predicted σ^I^-dependent promoters^10^ with each of the *C. thermocellum* σ^I^-factors (i.e., σ^I1^ -σ^6^) in a heterologous *B. subtilis* host system (Fig. 1B). The members of the promoter library were previously fused to a lacZ reporter gene^10^. In the present work, the activation of the reporter was tested in 24-well cell culture plates with Spizizen’s minimal medium using 5-bromo-4-chloro-3-indolyl-β-D-galactopyranoside (X-gal) as substrate (Fig. 1B). During the analysis, *C. thermocellum* σ^I5^ did not recognize any of the predicted promoters. As shown in Fig. 1B, with few exceptions, the majority of the σ^I^-dependent promoters are specific for their own σ^I^ factor. Interestingly, the major crosstalk between σ^I^s occurs in the two most important cellulosomal genes, *cipA* and *cel48S* (Fig. 1B). These genes encode the primary scaffoldin of the cellulosome, CipA^11,18,25^, and the most abundant enzyme in the cellulosome, Cel48S^6,26,27^, respectively.

### σ^I^-dependent promoters are structured into three motifs

**Figure 2:**
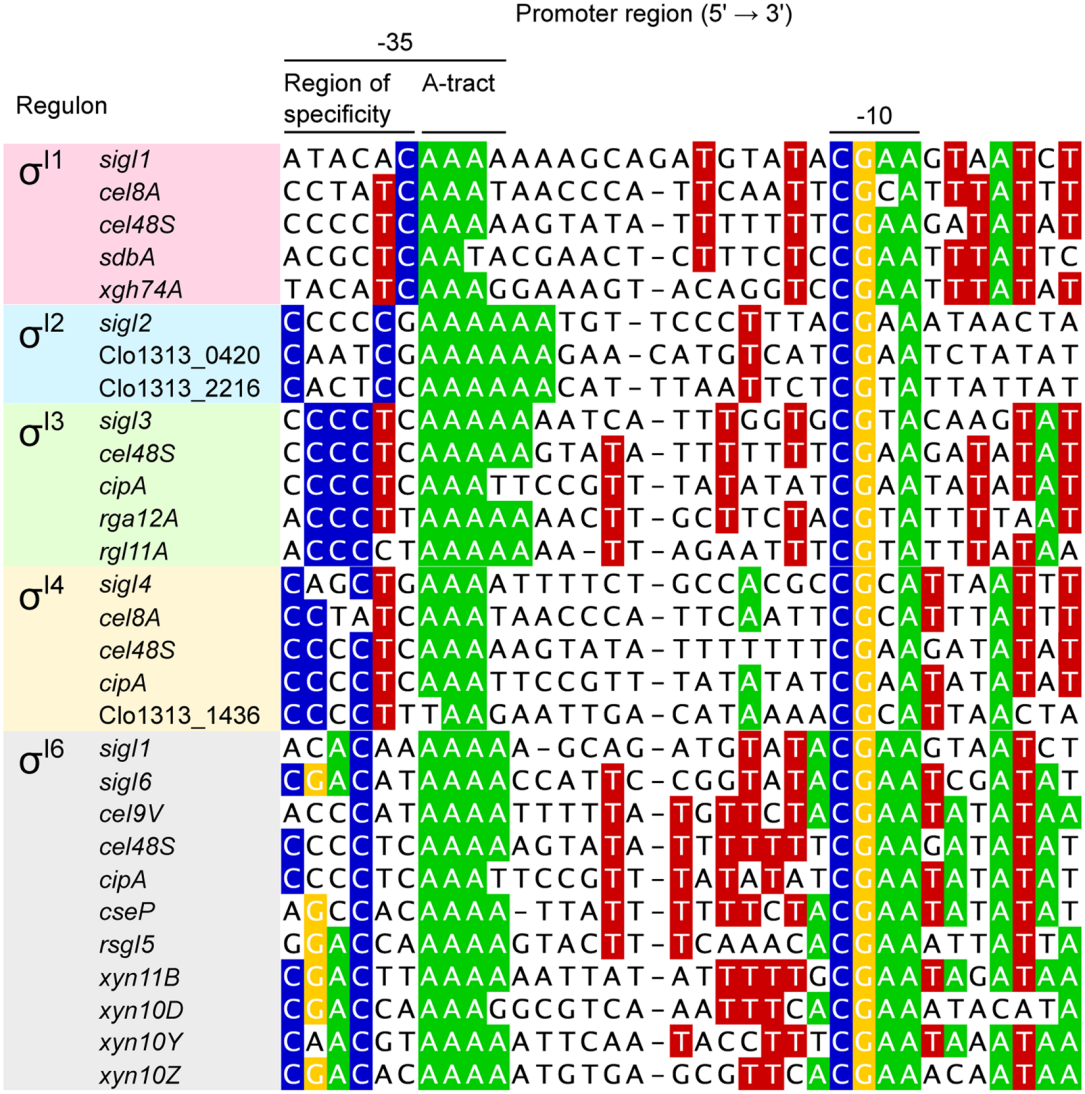
Dissection of *C. thermocellum* σ^I^-dependent promoters. The conservation of nucleotides shown in the alignment is divided into σ^I1^-, σ^I2^-, σ^I3^-, σ^I4^-, and σ^I6^-dependent promoters. The promoter sequences of each σ^I^ regulon were taken from Fig. 1A. The most conserved nucleotides of each σ^I^ regulon are highlighted.

According to the consensus promoter sequence, generated with the σ^I^-dependent promoters that were experimentally confirmed (Fig. 1), we divided the σ^I^-dependent promoters into three regions: (i) a highly conserved CGAA tetrad in the −10 element, (ii) a homopolymeric AAAA tetrad, herein termed the “A-tract motif” at the 3′ end of the −35 element, and (iii) a divergent region upstream of the A-tract motif. The most highly conserved promoter motifs, i.e., the A-tract in the −35 element and the CGAA tetrad in the −10 element, are proposed to be implicated in the “general” recognition of promoters by σ^I^s in cellulosome-producing bacteria (Fig. 1A). We suggest that the least conserved region, upstream of the A-tract motif in the −35 element, is implicated in the specificity of the different σ^I^ factors. Hence, we termed this divergent region as “region of specificity” (Fig. 1A). The alignments generated with the promoter sequences of each σ^I^ regulon support this observation (Fig. 2). A comparison of the region of specificity shows how each σ^I^ factor has different preferences. For example, whereas *C. thermocellum* σ^I1^ favors promoters with a CTC triad immediately upstream of the A-tract motif, *C. thermocellum* σ^I3^ prefers a CCC triad two nucleotides upstream of the A-tract motif (Fig. 2).

### Site-directed mutagenesis analysis confirmed the three motifs of σ^I^ promoters

The high stringency of promoter recognition by alternative σ^I^ factors was analyzed using *C. thermocellum* σ^I3^, because it recognizes highly conserve promoters with a characteristic C triad upstream of the A-tract motif in the −35 promoter element (Fig. 2). First, in order to verify the σ^I3^-dependent promoters, we mapped the TSSs of *rgl11A* and *rga12A* by the 5′-RACE technique (Supplementary Fig. S1). Next, the crucial nucleotides for recognition of *C. thermocellum* σ^I^-dependent promoters were analyzed by site-directed mutagenesis experiments using the σ^I3^-dependent promoter of *rgl11A*. The σ^I3^-dependent promoter of *rgl11A* was selected for the analysis because previous work showed that this promoter presented the highest activation by the *C. thermocellum* σ^I3^ factor^10^.

A promoter library with single transversion mutations (A to T or T to A, and C to G or G to C) in both −35 and −10 promoter elements was created. Additional transversion mutations were created upstream and downstream of both −35 and −10 promoter elements, replacing less conserved nucleotides. Subsequently, the promoter library was fused to a *lacZ-gfp* reporter operon. The promoter activities were thus studied in the heterologous *B. subtilis* host system. Quantification of promoter activities was performed by measuring GFP fluorescence, and the fluorescence of the different promoter variants was compared to that of the wild-type *rgl11A* promoter.

**Figure 3:**
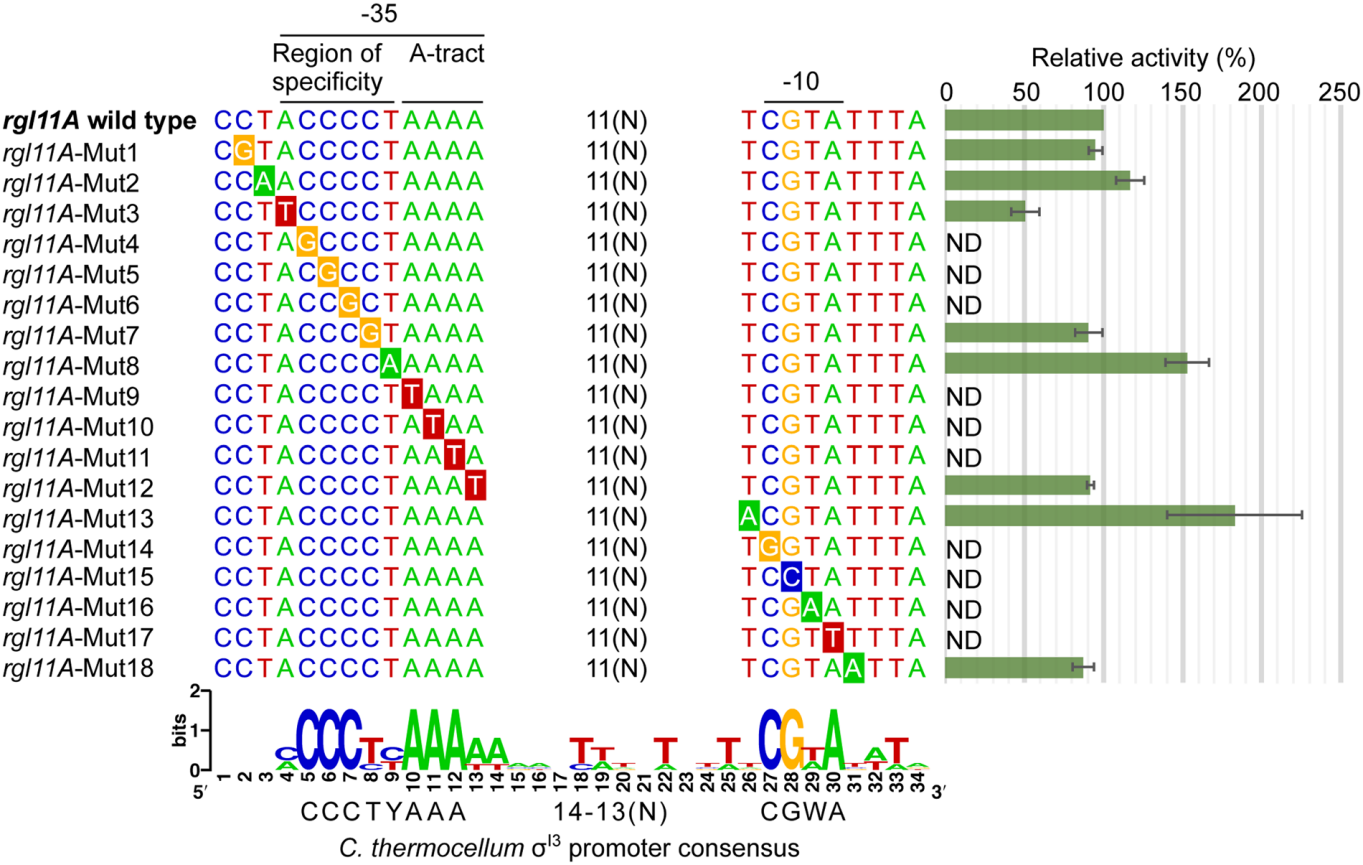
Evaluation of the validity of the σ^I^ promoter motifs by mutagenesis. The *rgl11* *A* σ^I3^-dependent promoter was selected for this analysis, because it presented the highest activation by the *C. thermocellum* σ^I3^ factor^10^. The WebLogo was generated with σ^I3^-dependent promoter sequences (Fig. 1A) and is shown to illustrate the effect of mutations in the most conserved nucleotides of the three motifs: (i) the −10 promoter element, (ii) the A-tract motif and (iii) the region of specificity. Mutations are indicated by highlighted nucleotides, and the effect of each mutation is shown as relative activity, compared to that of the wild-type control promoter *rgl11A*, defined as 100%. The relative activity shown is the average of three independent experiments. Predicted −35 and −10 promoter elements are indicated by lines above the *rgl11A* promoter sequences. The nucleotide code Y represents C or T, W represents A or T, and N represents any nucleotide. ND means not detected.

As shown in Fig. 3, mutations in the most conserved nucleotides in both −35 and −10 promoter elements have a negative effect, abolishing the detection of the GFP fluorescence. Mutations in less conserved nucleotides have a negligible effect, showing promoter activities at the same level as that of the wild-type promoter. Interestingly, the mutation from T to A between the highly conserved CCC and AAA triads of the −35 promoter element (*rgl11A*-Mut8; sequence underlined in CCCCTAAA) increased the fluorescence by 53%. As can be observed in the WebLogo shown in Fig. 3, *C. thermocellum* σ^I3^ apparently prefers promoter sequences enriched in adenines downstream of the −35 promoter element. This observation can explain the rise in activity identified in the promoter version of *rgl11A*-Mut8.

### σ^I^ factor recognizes highly stringent promoter sequences

To confirm the implication of the region of specificity in the stringency of σ^I^-dependent promoters, we searched for σ^I^-dependent promoters in the genome of *P. cellulosolvens* that resemble those of the *C. thermocellum* σ^I3^-dependent promoters. *P. cellulosolvens* was chosen for further comparison, because it produces the most complex cellulosome known to date^23^, and a previous report showed that as the cellulosome becomes more elaborate, the bacterium harbors more σ^I^ factors^11^. This observation was confirmed by our analysis which revealed that *P. cellulosolvens* genome encodes 16 σ^I^ paralogues (Supplementary Table S4), making this bacterium an excellent model for the study of multiple σ^I^ factors.

For the identification of putative σ^I^-dependent promoters in *P. cellulosolvens*, we first analyzed the upstream regions of all predicted σ^I^ genes (Supplementary Table S4). The search for promoters upstream of *P. cellulosolvens* σ^I^ genes was performed taking into account that the AAA triad and the CG dyad in the −35 and −10 promoter regions, respectively, are the most conserved nucleotides of the σ^I^-dependent promoters of cellulosome-producing bacteria (Fig. 1). The predicted putative σ^I^-dependent promoters of *P. cellulosolvens* σ^I^ genes are shown in Supplementary Table S4.

In order to predict putative σ^I^-dependent promoters, the genome sequence of *P. cellulosolvens* was analyzed using the same promoter motifs that were employed during the analysis of the upstream region of σ^I^ genes (AAA in the −35 region and CG in the −10 region). To delimit the promoter search, we included A, T or C downstream of the CG dyad in the −10 region (CGHH, where H represents A, T or C), because the predicted promoters of σ^I^ genes contain these nucleotides (Supplementary Table S4). The spacing between the −10 and −35 promoter elements was allowed to be between 12 to 15 bases. During this analysis, a collection of 140 σ^I^-dependent promoters were predicted (Supplementary Table S5).

**Figure 4:**
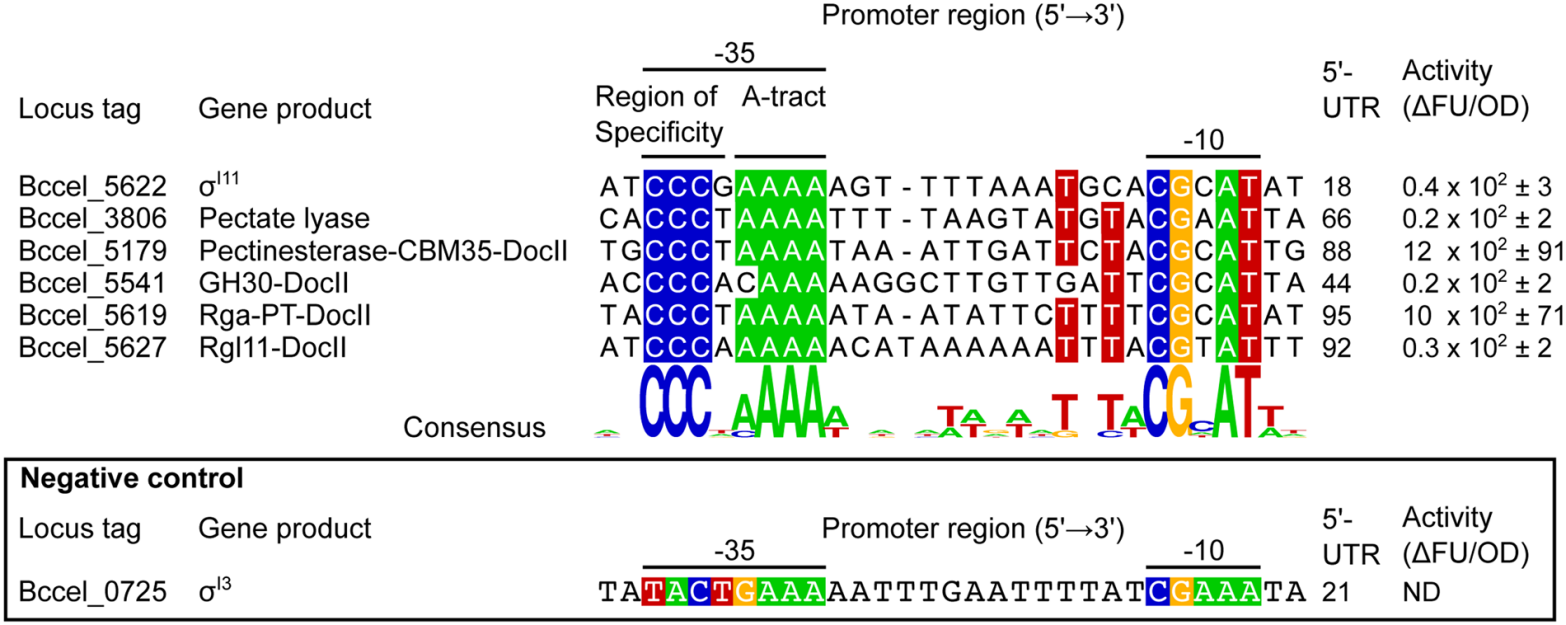
Alignment of *P. cellulosolvens* σ^I11^-dependent promoter sequences and quantitative evaluation of their recognition by the *P. cellulosolvens* σ^I11^ factor. The WebLogo was generated with the sequence shown in the alignment. Distances between the promoter region sequences used for the alignment and the first codon of corresponding genes are shown in the 5′-UTR (5′-untranslated region) column. Promoter activity was measured by quantifying the fluorescence (average of three independent experiments). Fluorescence units (FU) were calculated when cells reached an optical density (600 nm) of 1. ΔFU represents the activity of the induced promoter after subtracting the fluorescence obtained under uninduced conditions. ND (not detected) was assigned where ΔFU was negative, zero or the standard deviation exceeded ΔFU. As a negative control, the predicted σ^I^-dependent promoter of the *P. cellulosolvens* σ^I3^ gene was used. CBM, carbohydrate-binding module; Doc, dockerin; GH, glycoside hydrolase; Rga, rhamnogalacturan acetylesterase; PT, Pro-Thr repeat (pfam04886); Rgl, rhamnogalacturonan lyase.

Subsequently, in order to find putative *P. cellulosolvens* σ^I^-dependent promoter sequences resembling those of *C. thermocellum* σ^I3^-dependent promoters, the collection of predicted σ^I^-dependent promoters of *P. cellulosolvens* was analyzed by searching for cytosine enrichments in the region of specificity in the −35 promoter element. This analysis allowed the identification of one putative promoter upstream of *P. cellulosolvens sigI11* and five putative promoters upstream of genes encoding saccharolytic enzymes (Bccel_3806, Bccel_5179, Bccel_5541, Bccel_5619 and Bccel_5627). The alignment of the *P. cellulosolvens* predicted promoters is shown in Fig. 4. The highly conserved CCC triad can be observed in the region of specificity immediately upstream of the A-tract motif in the −35 promoter element. Additionally, the highly conserved CGCAT pentad in the −10 promoter element can also be observed. The majority of the *P. cellulosolvens* predicted promoters correspond to genes encoding catalytic modules which are probably involved in pectin degradation, such as pectate lyase, pectin esterase, Rga and Rgl [Fig. 4; ref.^28–30^].

Finally, to test the ability of *P. cellulosolvens* σ^I11^ to recognize the predicted promoters shown in Fig. 4, we fused the predicted promoters to a *gfp-lacZ* reporter operon. To overcome the lack of genetic tools in *P. cellulosolvens*, the promoter activities were also studied in a heterologous *B. subtilis* host system^10^. The recognition of the predicted promoters by *P. cellulosolvens* σ^I11^ was analyzed by measuring GFP fluorescence. As shown in Fig. 4, all the predicted promoters were recognized by *P. cellulosolvens* σ^I11^, suggesting that this alternative σ factor is likely involved in the regulation of genes encoding pectin-degrading enzymes, similar to its orthologous *C. thermocellum* σ^I3^ (Fig. 1B).

## Discussion

Cellulolytic clostridia produce one of the most efficient systems to degrade plant-cell wall polysaccharides — the multi-enzyme cellulosome complex^31^. The genomes of this group of bacteria contain dozens of genes, which encode different types of carbohydrate-active hydrolyzing enzymes and structural scaffoldin subunits, whose regulation is fine-tuned during the hydrolysis of biomass^32^. Although the cellulolytic capacities of cellulosome-producing bacteria have been the subject of study for many years^33–35^, the regulatory mechanisms that govern these processes are poorly known. Here, we show how a collection of five alternative σ^I^ factors in *C. thermocellum*, namely σ^I1^, σ^I2^, σ^I3^, σ^I4^ and σ^I6^, regulate the expression of 17 genes encoding different cellulosomal components (Fig. 1). This analysis reveals for the first time a sophisticated regulatory network of several alternative σ factors, which together control the enzymatic composition of the cellulosome (Fig. 1). Our results show how each σ^I^ factor has a particular regulon (Fig. 2) that correlates with its corresponding anti-σ^I^ factor that selectively senses a target polysaccharide^8,36,37^. Furthermore, with the results collected in *C. thermocellum*, we were able to predict the putative regulon of the *P. cellulosolvens* σ^I1^ factor (Fig. 4) and provide experimental evidence, for the first time in *P. cellulosolvens*, of the regulation of genes encoding cellulosomal components by an alternative σ factor (Fig. 4).

Alternative σ^I^ factors are a subfamily of the σ^70^ family which are unique, because not all σ^I^ factors are involved in the regulation of genes encoding saccharolytic enzymes or cellulosomal components. *B. subtilis* has only one σ^I^ factor that is induced by heat-shock^12^ and is involved in the maintenance of cell envelope integrity and homeostasis^38^. These observations show how σ^I^s are malleable alternative σ factors that have been adapted in different bacteria to perform various functions. However, during the course of evolution, when σ^I^ genes were extensively duplicated in the genome of cellulolytic clostridia to regulate the enzymatic composition of the cellulosome, their dependent promoter sequences were specialized in order to avoid regulatory overlap.

In the current work, three important observations support the hypothesis that alternative σ^I^ factors recognize stringent promoter sequences to discriminate among dozens of promoters that share similar motifs. First, each σ^I^ factor involved in the regulation of genes encoding saccharolytic enzymes and cellulosomal components in *C. thermocellum* has specific regulons with characteristic promoter sequences (Fig. 2). Second, mutagenesis analysis performed with the σ^I3^-dependent promoter of *C. thermocellum rgl11A* revealed a drastic reduction in promoter activity when the most conserved nucleotides were mutated (Fig. 3). Finally, the *C. thermocellum* σ^I3^ promoter consensus sequence allowed the identification of genes encoding putative pectin-degrading enzymes in another cellulolytic clostridium, *P. cellulosolvens* (Fig. 4).

Our results demonstrate that the region located upstream of the A-tract motif in the −35 promoter element has a crucial role for the discrimination of σ^I^-dependent promoters by the different σ^I^ factors (Figs 1 and 2). In general, all σ^I^ factors recognize promoters with a CGAA tetrad and an A-tract motif in the −10 and −35 promoter elements, respectively (Figs 1 and 2). However, the region upstream of the A-tract motif is characteristic to each σ^I^ regulon (Fig. 2). Indeed, this region, herein named region of specificity, was used to identify σ^I^-dependent promoters of genes involved in pectin degradation in the genome of *P. cellulosolvens* (Fig. 4).

The key role of the −35 promoter element for the recognition of σ^I^ factors is also supported by the conservation of homopolymeric A-tract motifs (Fig. 1). Intriguingly, in contrast to the A-tract motifs of σ^I^-dependent promoters, a recent report demonstrated in *B. subtilis* that a homopolymeric T-tract motif contributes to the activation of several ECF σ factors^13^. The latter T-tract motif is located downstream of the highly conserved AAC triad in the −35 promoter element of ECF σ factors. Here, the relevance of the A-tract motif was demonstrated during the mutagenesis analysis of the σ^I3^-dependent promoter of *C. thermocellum rgl11A*. We demonstrated how single mutations in the AAA triad of the −35 promoter element destroyed the recognition of the promoter by *C. thermocellum* σ^I3^ (Fig. 3). Furthermore, the addition of an extra A, by mutating the T located immediately upstream of the A-tract motif (i.e., *rgl11A*-Mut8; sequence underlined in CCCCTAAA) increased the strength of the promoter, providing promoter activity 53% higher than that of the wild-type promoter (Fig. 3).

It is also interesting that both T-tract and A-tract motifs are located in the same position in the −35 promoter region of ECF σ- and σ^I^-dependent promoters, respectively [ref.^13^ and Fig. 1, respectively]. Hence, we propose that this motif may help avoid crosstalk between ECF σs and σ^I^s. In this context, the respective −10 promoter elements of ECF σ- and σ^I^-dependent promoters are highly similar [ref.^13^ and Fig. 1, respectively]. The discrimination of promoters is critical in cellulolytic clostridia that harbor multiple σ^I^ factors, not only to avoid crosstalk between the different σ^I^s, but also to discriminate between the promoters of other types of alternative σ factors, such as the ECF σs. According to the Microbial Signal Transduction database (MiST_2.2_, http://mistdb.com/), the genome of *C. thermocellum* encodes 7 ECF σs, and our analysis revealed that the *P. cellulosolvens* genome encodes at least 22 ECF σs (Supplementary Table S6). Therefore, the possibility of having ECF σ- and σ^I^-dependent promoters with similar −35 regions is very high. Consequently, the A-tract motif of σ^I^-dependent promoters may also serve to avoid their recognition by ECF σ factors. This idea is further supported by the observation that none of the σ^I^-dependent promoters, which have been tested in our laboratory by using the *B. subtilis* heterologous host cell system (that is devoid of its native σ^I^/RsgI system), were activated by the resident *B. subtilis* σ factors^10,11^.

It is worth mentioning that genomic context may also play a defined role in promoter selectivity. It has been reported that some important sequences which reside outside of the classic −35 and −10 promoter elements can be implicated in the recognition of the promoter^39,40^. For example, most of the promoters that are dependent on the *Escherichia coli* and *Salmonella enterica* σ^E^, an ECF σ factor, require a sequence upstream of the −35 promoter element (UP-element) to increase their strength^39^. Likewise, promoters that are dependent on σ^I^ factors may also require UP-elements. The presence of motifs that reside outside of the classic −35 and −10 promoter elements that help to increase the strength of the promoter, or compensate for a poor −35 or −10 promoter element that deviates from consensus, is a phenomenon known as “mix and match”^40–42^. Future identification of σ^I^-dependent promoters may be improved by taking into account mix-and-matching as a promoter recognition mechanism, thereby generating a better understanding of the biological function of each σ^I^ factor.

It is also important to note that, although the *C. thermocellum* σ^I^ factors have a defined regulon with little crosstalk, the major regulatory overlap between σ^I^s occurs in the two most important cellulosomal genes, *cipA*^11,18,25^ and *cel48S*^6,26,27^ (Fig. 1B). Close inspection of the σ^I^-dependent promoter of *C. thermocellum cipA* and *cel48S* reveals that both −35 and −10 promoter regions are identical, with a CCCCTCAAA nonad and a CGAA tetrad, respectively (Fig. 1A). Additionally, the σ^I^-dependent promoter of *C. thermocellum cipA* and *cel48S* have a conserved AT dyad, three nucleotides downstream of the CGAA tetrad in the −10 promoter element that is present in nearly all of the σ^I^-dependent promoters shown in Fig. 1A. These observations suggest that the σ^I^-dependent promoter of *C. thermocellum cipA* and *cel48S* may represent a type of “universal” promoter that is used by the bacterium to assure expression of relevant genes.

Previous works have shown that both CipA and Cel48S are fundamental components of the cellulosome^25–27^. Hence their expression should be assured in the presence of a wide number of polysaccharide substrates and conditions^6,27^. In this sense, it would seem logical to use more than one alternative σ factor to regulate their expression. However, the recognition of specific promoter sequences by each of the regulators would be the consequence of a long and complex evolutionary process. A more practical approach to avoid this process is the utilization of a single “universal promoter” such as the one presented in this work. From an evolutionary point of view, it is very likely that the common ancestor of these σ^I^ factors recognized a similar promoter sequence to that used by *cipA* and *cel48S*. Later, each of the duplicated genes evolved to encode specialized versions of the σ^I^s capable of recognizing unique promoters, thus reducing unnecessary crosstalk and limiting their regulons while maintaining regulatory overlap of these critical components.

Interestingly, the σ^I^-dependent promoters of *C. thermocellum sigI3* harbor almost the same promoter elements present in *cipA and cel48S* σ^I^-dependent promoters. In contrast, the σ^I^-dependent promoters of *C. thermocellum sigI3* are only recognized by σ^I3^ (Fig. 1B). The difference is present in the −10 promoter element. Whereas the σ^I^-dependent promoter of *cipA* and *cel48S* have the CGAA tetrad in the −10 element, the σ^I^-dependent promoter of *sigI3* has the CGTA tetrad. Additionally, the conserved AT dyad, downstream of the −10 element that is present in the σ^I^-dependent promoter of *cipA* and *cel48S* (Fig. 1A), is less conserved in the promoter of *sigI3*, since it has a GT dyad in the same position (Fig. 1A). Thus, these small changes in the −10 promoter element can also help to improve specificity and avoid regulatory overlap.

Direct analysis of the multiple σ^I^ factors in *C. thermocellum* represents a serious challenge, owing to the regulatory overlap between the different σ^I^ factors with some of the important genes, such as *cipA* and *cel48S* (Fig. 1). Moreover, the genome of *C. thermocellum* may harbor additional as-yet-unidentified σ^I^-dependent promoters that may be activated by more than one σ^I^ factor. Therefore, during future studies of a particular σ^I^ factor directly in *C. thermocellum*, we could expect a cascade of interactions among the different σ^I^s. Additionally, cellulosomal component genes can also have σ^A^-dependent promoters^11,43^ and other regulatory proteins^44^, making the analysis of σ^I^s directly in *C. thermocellum* more complex. If these observations are not taken into account during the direct analysis of σ^I^s in *C. thermocellum*, the results obtained can be misinterpreted. These observations apply also for other cellulolytic clostridia with multiple σ^I^s, such as *P. cellulosolvens* (Supplementary Table S4). Hence, as shown in the present report, the application of the heterologous *B. subtilis* host system for analysis of the multiple σ^I^ factors of cellulolytic clostridia is advantageous. The predicted σ^I^-dependent promoters can be experimentally tested in *B. subtilis* and corroborated *in vivo* by mapping the TSSs of their associated genes (Supplementary Fig. S1).

In conclusion, in the present work, we show how the employment of classical microbiology genetic tools, such as the *LacZ* reporter system^45^, together with the well-known Gram-positive bacterium *B. subtilis* as heterologous host^10^, enabled us to decipher the regulatory networks of the multiple alternative σ^I^ factors, of one of the most efficient and most intricate cellulolytic systems in nature – the bacterial cellulosome.

## Methods

### Bacterial strains, growth media and culture conditions

*C. thermocellum* DSM 1313 and *P. cellulosolvens* DSM2933 were obtained from the DSMZ (German Collection of Microorganisms and Cell Cultures, Braunschweig, Germany). The recognition of predicted σ^I^-dependent promoters by *C. thermocellum* and *P. cellulosolvens* σ^I^ factors was analyzed in a heterologous *B. subtilis* host system that was developed in a previous work^10^. The *B. subtilis* strains constructed used in this work (Supplementary Table S1) are isogenic derivatives of the *B. subtilis* strain CO02 that is devoid of its *sigI-rsgI* operon^10^.

*C. thermocellum* and *P. cellulosolvens* were grown using the media and condition described by the ref.^9^ and the DSMZ, respectively. *B. subtilis* and *E. coli* were cultivated routinely on solid LB Broth (Lennox, Difco, BD Diagnostics, Maryland, USA) or in liquid LB Broth (at 250 rpm) at 37 °C. The expression of genes under the P_*xylA*_ promoter was induced with D-xylose using a final concentration of 10 g/L (Sigma-Aldrich). When required the following antibiotics (all from Sigma-Aldrich) were added at the indicated final concentration: ampicillin (100 µg/mL, Amp), kanamycin (50 µg/mL, Kan), chloramphenicol (5 µg/mL, Cam) or erythromycin (3 µg/mL, Erm).

### DNA manipulation techniques and construction of plasmids

The primers and plasmids used in the present work are listed in Tables S2 and S3, respectively. Plasmids were constructed by standard molecular cloning techniques using restriction enzymes and ligase, or by the ligase-independent cloning technique based on the In-Fusion HD Cloning Kit (Clontech Laboratories, Inc., California, USA).

The pAX01 integration vector was used to express the *C. thermocellum* and *P. cellulosolvens* σ^I^ factors in *B. subtilis*^46^. This plasmid has the xylose-inducible promoter P_*xylA*_ to control the expression of a gene of interest, integrates at *B. subtilis lacA* locus and harbors an *erm* cassette as a selectable marker. First, pAX01 was linearized with the restriction enzyme BamHI. Later, *P. cellulosolvens sigI11* was PCR-amplified using primers P1 and P2. Finally, the PCR product was cloned using the In-Fusion HD Cloning Kit into the linearized pAX01 vector, thereby obtaining the pAX01-Bc-SigI11 plasmid. To express the *C. thermocellum* σ^I1^, σ^I2^, σ^I3^, σ^I4^, σ^I5^ and σ^I6^ factors in *B. subtilis* we used the pAX01 derived plasmids, pAX01-SigI1, pAX01-SigI2, pAX01-SigI3, pAX01-SigI4, pAX01-SigI5 and pAX01-SigI6, that were constructed in previous works^10,11^.

The regulons of *C. thermocellum* σ^I1^, σ^I2^, σ^I3^, σ^I4^, σ^I5^ and σ^I6^ were analyzed by using the 40 putative σ^I^-dependent promoters that were predicted in a previous work^10^. With the exception of the putative predicted σ^I^-dependent promoters of *C. thermocellum cel48S*, we used a library of the previously predicted promoters that was fused to the LacZ reporter gene of the pBS1C-LacZ integration vector in a previous work^10^. pBS1C-LacZ integrates at the *B. subtilis amyE* locus and carries a *cam* resistance cassette as a selectable marker^47^. In the case of the putative predicted σ^I^-dependent promoters of *C. thermocellum cel48S*, its sequence was PCR-amplified using primers P3 and P4. Subsequently, the PCR product was digested with the restriction enzymes EcoRI and BamHI and fused to the LacZ reporter gene of pBS1C-LacZ that was previously cut with the same restriction enzymes, thereby obtaining the pProm-Ct-Cel48S derived plasmid.

In order to study the important nucleotides for the recognition of σ^I^-dependent promoters, we used the pBS1C-GFP-LacZ integration vector which harbors a promoterless *gfp-lacZ* reporter operon^11^. pBS1C-GFP-LacZ integrates at the *B. subtilis amyE* locus and carries a *cam* resistance cassette as a selectable marker^11^. The analysis of promoter recognition was performed with mutant versions of the σ^I3^-dependent promoter of *C. thermocellum rgl11A* that were created by site-directed mutagenesis and fused to the *gfp-lacZ* reporter operon of pBS1C-GFP-LacZ. To introduce individual mutations in both −35 and −10 promoter regions, the reverse primers from P5 to P22, which contain the mutated nucleotide, were used with the forward primer P23. In order to compare the mutant versions, a wild type version of the *rgl11A* σ^I3^-dependent promoter was PCR-amplified using the primer pair P23-P24. After PCR amplification of the promoter mutant versions, the PCR products were digested with the restriction enzymes EcoRI and BamHI. Finally, each digested PCR product was cloned into pBS1C-GFP-LacZ that was digested previously with the same restriction enzymes, thereby obtaining the pBS1C-GFP-LacZ derived plasmids listed in Supplementary Table S3.

The recognition of σ^I^-dependent promoters by *P. cellulosolvens* σ^I11^ was analyzed with the pBS1C-GFP-LacZ integration vector. The predicted σ^I^-dependent promoters of Bccel_5622 (*P. cellulosolvens* σ^I11^ gene), Bccel_3856, Bccel_5179, Bccel_5541, Bccel_5619 and Bccel_5627 were PCR-amplified using the primer pairs P25-P26, P27-P28, P29-P30, P31-P32, P33-P34 and P35-P36, respectively. Later, each PCR product was digested with the restriction enzymes EcoRI and BamHI, except the promoter of Bccel_5541 that was PCR-amplified for cloning with the In-Fusion system. Finally, each digested PCR product was cloned into the pBS1C-GFP-LacZ plasmid that was digested previously with the same restriction enzymes, thereby obtaining the pBS1C-GFP-LacZ derived plasmids listed in Supplementary Table S3 (plasmid #26 to #30). In the case of the predicted promoter of Bccel_5541, its DNA sequence was cloned into pBS1C-GFP-LacZ (previously linearized with EcoRI and BamHI) using the In-Fusion HD Cloning Kit (Supplementary Table S3, plasmid #31).

### Mapping of the TSSs

In order to map the TSSs we purified total RNA of *C. thermocellum* following the protocols described in a previous publication^11^. The mRNA 5′-ends were mapped using the 5′-RACE technique with the SMARTer® RACE 5′/3′ kit (Clontech) according to supplier protocols. Briefly, total RNA was subjected to RT-PCR with random primers and the SMARTer II oligonucleotide. Subsequently the 5′-RACE-Ready cDNA was submitted to a PCR amplification using the Universal Primer A Mix (a combination of Universal Primer Long and Universal Primer Short) and the gene specific primer (P40, P41, P42, P43 or P44 for *sigI2*, *sigI3*, *sigI4*, *rgl11A* and *rga12A*, respectively; Supplementary Table S2). Then, this PCR-product was subjected to a second PCR with the Universal Primer Short and the nested gene specific primer (P45, P46, P47, P48 or P49 for *sigI2*, *sigI3*, *sigI4*, *rgl11A* and *rga12A*, respectively; Supplementary Table S2). Finally, the PCR-products were gel purified, cloned into pRACE using the In-Fusion® HD Cloning kit and sequenced.

### Construction of *B. subtilis* strains and analysis of promoter activities

*B. subtilis* was transformed by using the natural competence method^48^. Chromosomal integration of plasmids by a double-crossover event was confirmed by colony PCR using the primers listed in Tables S2. To analyze the LacZ reporter system, *B. subtilis* strain samples were taken from the −80 °C glycerol stock, inoculated in LB broth (3 mL) with Cam and grown overnight. The next day, the cells were centrifuged 15 min at 3000 × g and resuspended in 1 mL of Spizizen’s minimal media^48^ with fructose (1.8 g/L final) as the carbon source. Finally, the cells were incubated in 24-well cell culture plates at 150 rpm. To observe the LacZ activity, the Spizizen’s minimal media was supplemented with X-gal (40 mg/L final). To measure the fluorescence associated to the GFP reporter system, we followed the protocol described elsewhere^11^. Fluorescence units (FU) were calculated when the cells reached an optical density at 600 nm of 1 and represent the activity of the induced promoter after subtracting the values obtained under uninduced conditions, i.e. ΔFU = FU_induced cells_ − FU_uninduced cells_.

### Bioinformatics

Promoter motifs searches were carried out with the Pattern Locator program^49^. The analysis of the promoter motif sequences was performed with the Jalview software^50^. Multiple sequence alignment (MSA) of promoter sequences was performed using the T-Coffee algorithm^51^ implemented by Jalview. DNA sequence logos were generated with the program WebLogo^52^.

### Data availability

All data generated or analysed during this study are included in this published article (and its Supplementary Information files).

## Electronic supplementary material

Supplementary information
