# Supplementary material for: Regulation of biomass degradation by alternative σ factors in cellulolytic clostridia
Source: Sci Rep. 2018 Jul 23;8:11036. doi: 10.1038/s41598-018-29245-5 (PMC6056542; doi:10.1038/s41598-018-29245-5)
Supplement: Supplementary file 1 — Supplementary information [file 41598_2018_29245_MOESM1_ESM.pdf]

Regulation of biomass degradation by alternative σ factors in cellulolytic clostridia

Lizett Ortiz de Ora[1], Raphael Lamed[1], Ya-Jun Liu[2], Jian Xu[2], Qiu Cui[2], Yingang Feng[2], Yuval Shoham[3], Edward A. Bayer[4], Iván Muñoz-Gutiérrez[4,#,*]

Supplementary Information

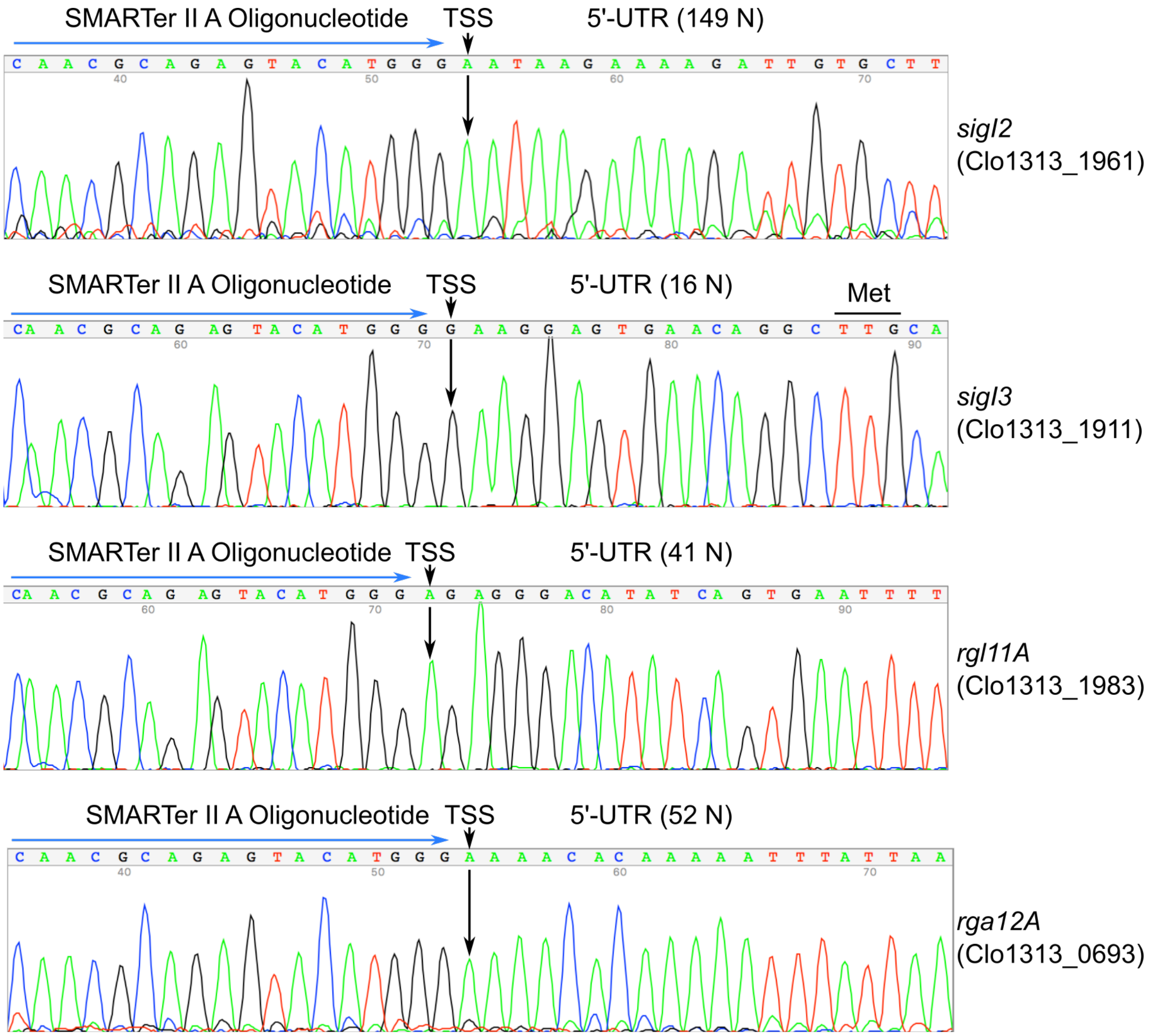

Figure S1. Identification of TSSs associated to σ^I-dependent promoters by the 5'-RACE technique. The TSSs are indicated by the vertical black arrows. Promoter sequences associated with each of the mapped TSSs are shown in Figure 1A. In some chromatograms, the first codon of the corresponding gene can be observed, and they are indicated by the amino acid Met. The sequence below the horizontal blue arrow represents the partial sequence of the SMARTer II A Oligonucleotide. N represents any nucleotide. 5'-UTR, 5'-untranslated region.

Table S1. ***Bacillus subtilis*** **strains[a] used in the present work.**

**Strain derivation and name**	**Relevant genotype[b]**	**σ factor or promoter harbored**
From pAX01		
SCt3[c]	CO02 *lacA*::(P_{xyl}-*sigI3*$_{Ct}$ *erm*)	*C. thermocellum* σ^{I3}
SBc11	CO02 *lacA*::(P_{xyl}-*sigI11*$_{Bc}$ *erm*)	*B. cellulosolvens* σ^{I11}
From pAX01 and pBS1C-GFP-LacZ		
SCt3-Prgl11A	CO02 *lacA*::(P_{xyl}-*sigI3*$_{Ct}$ *erm*) *amyE*::(P_{rgl11A_Ct}-*gfp-lacZ cat*)	Wild type and mutant versions of the σ^{I3}-dependent promoter of *C. thermocellum rgl11A* used to analyze the validity of the *C. thermocellum* σ^{I3} promoter consensus sequence
SCt3-Prgl11A-Mut1	CO02 *lacA*::(P_{xyl}-*sigI3*$_{Ct}$ *erm*) *amyE*::($P_{rga11A\text{-}Mut1\_Ct}$-*gfp-lacZ cat*)	
SCt3-Prgl11A-Mut2	CO02 *lacA*::(P_{xyl}-*sigI3*$_{Ct}$ *erm*) *amyE*::($P_{rgl11A\text{-}Mut2\_Ct}$-*gfp-lacZ cat*)	
SCt3-Prgl11A-Mut3	CO02 *lacA*::(P_{xyl}-*sigI3*$_{Ct}$ *erm*) *amyE*::($P_{rgl11A\text{-}Mut3\_Ct}$-*gfp-lacZ cat*)	
SCt3-Prgl11A-Mut4	CO02 *lacA*::(P_{xyl}-*sigI3*$_{Ct}$ *erm*) *amyE*::($P_{rgl11A\text{-}Mut4\_Ct}$-*gfp-lacZ cat*)	
SCt3-Prgl11A-Mut5	CO02 *lacA*::(P_{xyl}-*sigI3*$_{Ct}$ *erm*) *amyE*::($P_{rgl11A\text{-}Mut5\_Ct}$-*gfp-lacZ cat*)	
SCt3-Prgl11A-Mut6	CO02 *lacA*::(P_{xyl}-*sigI3*$_{Ct}$ *erm*) *amyE*::($P_{rgl11A\text{-}Mut6\_Ct}$-*gfp-lacZ cat*)	
SCt3-Prgl11A-Mut7	CO02 *lacA*::(P_{xyl}-*sigI3*$_{Ct}$ *erm*) *amyE*::($P_{rgl11A\text{-}Mut7\_Ct}$-*gfp-lacZ cat*)	
SCt3-Prgl11A-Mut8	CO02 *lacA*::(P_{xyl}-*sigI3*$_{Ct}$ *erm*) *amyE*::($P_{rgl11A\text{-}Mut8\_Ct}$-*gfp-lacZ cat*)	
SCt3-Prgl11A-Mut9	CO02 *lacA*::(P_{xyl}-*sigI3*$_{Ct}$ *erm*) *amyE*::($P_{rgl11A\text{-}Mut9\_Ct}$-*gfp-lacZ cat*)	
SCt3-Prgl11A-Mut10	CO02 *lacA*::(P_{xyl}-*sigI3*$_{Ct}$ *erm*) *amyE*::($P_{rgl11A\text{-}Mut10\_Ct}$-*gfp-lacZ cat*)	
SCt3-Prgl11A-Mut11	CO02 *lacA*::(P_{xyl}-*sigI3*$_{Ct}$ *erm*) *amyE*::($P_{rgl11A\text{-}Mut11\_Ct}$-*gfp-lacZ cat*)	
SCt3-Prgl11A-Mut12	CO02 *lacA*::(P_{xyl}-*sigI3*$_{Ct}$ *erm*) *amyE*::($P_{rgl11A\text{-}Mut12\_Ct}$-*gfp-lacZ cat*)	
SCt3-Prgl11A-Mut13	CO02 *lacA*::(P_{xyl}-*sigI3*$_{Ct}$ *erm*) *amyE*::($P_{rgl11A\text{-}Mut13\_Ct}$-*gfp-lacZ cat*)	
SCt3-Prgl11A-Mut14	CO02 *lacA*::(P_{xyl}-*sigI3*$_{Ct}$ *erm*) *amyE*::($P_{rgl11A\text{-}Mut14\_Ct}$-*gfp-lacZ cat*)	
SCt3-Prgl11A-Mut15	CO02 *lacA*::(P_{xyl}-*sigI3*$_{Ct}$ *erm*) *amyE*::($P_{rgl11A\text{-}Mut15\_Ct}$-*gfp-lacZ cat*)	
SCt3-Prgl11A-Mut16	CO02 *lacA*::(P_{xyl}-*sigI3*$_{Ct}$ *erm*) *amyE*::($P_{rgl11A\text{-}Mut16\_Ct}$-*gfp-lacZ cat*)	
SCt3-Prgl11A-Mut17	CO02 *lacA*::(P_{xyl}-*sigI3*$_{Ct}$ *erm*) *amyE*::($P_{rgl11A\text{-}Mut17\_Ct}$-*gfp-lacZ cat*)	
SCt3-Prgl11A-Mut18	CO02 *lacA*::(P_{xyl}-*sigI3*$_{Ct}$ *erm*) *amyE*::($P_{rgl11A\text{-}Mut18\_Ct}$-*gfp-lacZ cat*)	
SBc11-PBccel_3806	CO02 *lacA*::(P_{xyl}-*sigI11*$_{Bc}$ *erm*) *amyE*::(P_{Bccel_3806}-*gfp-lacZ cat*)	σ^{I11}-dependent promoter of *B. cellulosolvens* used to analyze their activation by *B. cellulosolvens* σ^{I11}
SBc11-PBccel_5179	CO02 *lacA*::(P_{xyl}-*sigI11*$_{Bc}$ *erm*) *amyE*::(P_{Bccel_5179}-*gfp-lacZ cat*)	
SBc11-PBccel_5541	CO02 *lacA*::(P_{xyl}-*sigI11*$_{Bc}$ *erm*) *amyE*::(P_{Bccel_5541}-*gfp-lacZ cat*)	
SBc11-PBccel_5619	CO02 *lacA*::(P_{xyl}-*sigI11*$_{Bc}$ *erm*) *amyE*::(P_{Bccel_5619}-*gfp-lacZ cat*)	
SBc11-PBccel_5622	CO02 *lacA*::(P_{xyl}-*sigI11*$_{Bc}$ *erm*) *amyE*::(P_{Bccel_5622}-*gfp-lacZ cat*)	
SBc11-PBccel_5627	CO02 *lacA*::(P_{xyl}-*sigI11*$_{Bc}$ *erm*) *amyE*::(P_{Bccel_5627}-*gfp-lacZ cat*)	

[a] The B. subtilis strains constructed in the present work are isogenic derivatives of strain CO02 [1].

[b] *Ct*, *C. thermocellum*; *Bc*, *B. cellulosolvens*.

[c] Strain constructed in a previous work[1].

Table S2. Primers used in the present work.

#	Name	Sequence 5'→3'	Brief description
P1[a]	Fw.BcsigI11.IF	GGGGGAAATGGGATCATGCTATTTATATCAGCTGTTTTAA	Amplification of *Bc sigI11* gene during the construction of pAX01-Bc-SigI11
P2[a]	Rv.BcsigI11.IF	CGCGGGAGCTCGGATTTATCTAAATACCTGGTTTAAATATGC	
P3	Fw.cel48S.eco	TAGAATTCGCACAAGAACTTCAAATGTTTCC	Amplification of *Ct cel48S* promoter
P4	Rv.cel48S.bam	TAGGATCCTCAATAAATTCCATATATATATCTTCG	
P5	Rv.rgl11.mut1	GCGGATCCGTCCCTCTTTTTATAAATACGAAATTCTAATTTTT TTAGGGGTACGTTTTTC	Mutation from C to G in the -35 element (5' CTACCCCTAAAA 3' mutation in the underline nucleotide).
P6	Rv.rgl11.mut2	GCGGATCCGTCCCTCTTTTTATAAATACGAAATTCTAATTTTT TTAGGGGTTGGTTTTTC	Mutation from T to A in the -35 element. (5' CTACCCCTAAAA 3' mutation in the underline nucleotide).
P7	Rv.rgl11.mut3	GCGGATCCGTCCCTCTTTTTATAAATACGAAATTCTAATTTTT TTAGGGGAAGGTTTTTC	Mutation from A to T in the -35 element. (5' CTACCCCTAAAA 3' mutation in the underline nucleotide).
P8	Rv.rgl11.mut4	GCGGATCCGTCCCTCTTTTTATAAATACGAAATTCTAATTTTT TTAGGGCTAGGTTTTTC	Mutation from C to G in the -35 element. (5' CTACCCCTAAAA 3' mutation in the underline nucleotide).
P9	Rv.rgl11.mut5	GCGGATCCGTCCCTCTTTTTATAAATACGAAATTCTAATTTTT TTAGGCGTAGGTTTTTC	Mutation from C to G in the -35 element. (5' CTACCCCTAAAA 3' mutation in the underline nucleotide).
P10	Rv.rgl11.mut6	GCGGATCCGTCCCTCTTTTTATAAATACGAAATTCTAATTTTT TTAGCGGTAGGTTTTTC	Mutation from C to G in the -35 element. (5' CTACCCCTAAAA 3' mutation in the underline nucleotide).
P11	Rv.rgl11.mut7	GCGGATCCGTCCCTCTTTTTATAAATACGAAATTCTAATTTTT TTACGGGTAGGTTTTTC	Mutation from C to G in the -35 element. (5' CTACCCCTAAAA 3' mutation in the underline nucleotide).
P12	Rv.rgl11.mut8	GCGGATCCGTCCCTCTTTTTATAAATACGAAATTCTAATTTTT TTTGGGGTAGGTTTTTC	Mutation from T to A in the -35 element. (5' CTACCCCTAAAA 3' mutation in the underline nucleotide).
P13	Rv.rgl11.mut9	GCGGATCCGTCCCTCTTTTTATAAATACGAAATTCTAATTTTT TAAGGGGTAGGTTTTTC	Mutation from A to T in the -35 element. (5' CTACCCCTAAAA 3' mutation in the underline nucleotide).
P14	Rv.rgl11.m10	GCGGATCCGTCCCTCTTTTTATAAATACGAAATTCTAATTTTT ATAGGGGTAGGTTTTTC	Mutation from A to T in the -35 element. (5' CTACCCCTAAAA 3' mutation in the underline nucleotide).
P15	Rv.rgl11.mut11	GCGGATCCGTCCCTCTTTTTATAAATACGAAATTCTAATTTTA TTAGGGGTAGGTTTTTC	Mutation from A to T in the -35 element. (5' CTACCCCTAAAA 3' mutation in the underline nucleotide).
P16	Rv.rgl11.mut12	GCGGATCCGTCCCTCTTTTTATAAATACGAAATTCTAATTTAT TTAGGGGTAGGTTTTTC	Mutation from A to T in the -35 element. (5' CTACCCCTAAAA 3' mutation in the underline nucleotide).
P17	Rv.rgl11.mut13	GCGGATCCGTCCCTCTTTTTATAAATACGTAATTCTAATTTTT TTAGG	Mutation from T to A in the -10 element (5' TCGTAT 3' mutation in the underline nucleotide).

P18	Rv.rgl11.mut14	GCGGATCCGTCCCTCTTTTTATAAATACCAAATTCTAATTTTTTTAGG	Mutation from C to G in the -10 element (5' TCGTAT 3' mutation in the underline nucleotide).
P19	Rv.rgl11.mut15	GCGGATCCGTCCCTCTTTTTATAAATAGGAAATTCTAATTTTTTTAGG	Mutation from G to C in the -10 element (5' TCGTAT 3' mutation in the underline nucleotide).
P20	Rv.rgl11.mut16	GCGGATCCGTCCCTCTTTTTATAAATTCGAAATTCTAATTTTTTTAGG	Mutation from T to A in the -10 element (5' TCGTAT 3' mutation in the underline nucleotide).
P21	Rv.rgl11.mut17	GCGGATCCGTCCCTCTTTTTATAAAAACGAAATTCTAATTTTTTTAGG	Mutation from A to T in the -10 element (5' TCGTAT 3' mutation in the underline nucleotide).
P22	Rv.rgl11.mut18	GCGGATCCGTCCCTCTTTTTATAATTACGAAATTCTAATTTTTTTAGG	Mutation from T to A in the -10 element (5' TCGTAT 3' mutation in the underline nucleotide).
P23	Fw.rgl11	CAGAATTCATATGCCAACTGAATAATGGTGT	Amplification of *Ct rgl11* promoter
P24	Rv.rgl11	CAGGATCCAATTCACTGATATGTCCCTC	
P25	Fw.BcPsigI11.eco	GCGAATTCACATGGCGTTTTTTATATTATATC	Amplification of *Bc sigI11* predicted promoter
P26	Rv.BcPsigI11.bam	GCGGATCCCTATCACACCCTTGCTATATATG	
P27	Fw.BcP3806.eco	GAGAATTCGCAGAAGATGTGTTGTAAATTAC	Amplification of *Bc* Bccel_3806 predicted promoter
P28	Rv.BcP3806.bam	GCGGATCCAGTAAGTTACTCCCTAGGAGTC	
P29	Fw.BcP5179.eco	GAGAATTCAGCCTATCTTGAATGAAACATATATTTG	Amplification of *Bc* Bccel_5179 predicted promoter
P30	Rv.BcP5179.bam	GTGGATCCCACTTTCCATTTTGCAACAATC	
P31[a]	Fw.BcP5541.IF	TCAAACATGAGAATTCTAAACTTCATTATTTTATCGTATACC	Amplification of *Bc* Bccel_5541 predicted promoter
P32[a]	Rv.Bc5541.IF	GTTAATCAGCGGATCCTTTTGAATATAAGTGAATTCTAGAC	
P33	Fw.BcP5619.eco	GTGAATTCCACTTCCACTATAGTTTAATCTTC	Amplification of *Bc* Bccel_5619 predicted promoter
P34	Rv.BcP5619.bam	GCGGATCCCCGTAAGATGCAAGATCTCACGC	
P35	Fw.BcP5627.eco	GTGAATTCGTTATCAGAAAACTGGTAAAACCAC	Amplification of *Bc* Bccel_5627 predicted promoter
P36	Rv.BcP5627.bam	GCGGATCCCTATATTCTGCTATATAGTTTCTAGAC	
P37[b]	SMARTer II A Oligonucleotide	AAGCAGTGGTATCAACGCAGAGTACXXXXX	5'-RACE analysis
P38	Universal Primer Long	CTAATACGACTCACTATAGGGCAAGCAGTGGTATCAACGCAGAGT	
P39	Universal Primer Short	CTAATACGACTCACTATAGGGC	
P40	sigI2.gsp	TTGTGCTCCGTTCAATGTTGTCACCTCC	
P41	sigI3.gsp	GATTACGCCAAGCTTTCTCAAAGCTGACCAGTTCCTGCCTTACC	
P42	sigI4.gsp	CCTGCTCATCACTGTTTAACGCTGCCG	
P43	rgl11A.gsp	GATTACGCCAAGCTTTCTGACGCGCACCAGTAGGTGTAGGTCC	
P44	rga12A.gsp	GATTACGCCAAGCTTACTTGCCCTCGAAGTGTTTCCCAAAGTGAC	
P45	sigI2.ngsp	GGCAAGACCCACGCTGAACTCATCACTG	
P46	sigI3.ngsp	GATTACGCCAAGCTTGACCGCTGGCATCGGACATCAATAATCTTTCG	
P47	sigI4.ngsp	GTCCTACGCTGTATTCCTCGCTCACCTCTG	
P48	rgl11A.ngsp	GATTACGCCAAGCTTCCGTGCCCACCGGAAACTTGTCAATAAC	
P49	rga12A.ngsp	GATTACGCCAAGCTTGCCTGATGCCGCAACATCCCTCATATTC	
P50	GanQ-Cnf	ATATACATTGCCCGTCGGTC	

P51	Erm-Cnf	GCAATGAAACACGCCAAAG	Confirmation of chromosomal integration at *Bs lacA* locus. Primers bind to *ganQ* upstream of *lacA* (*ganA*), and to the *erm* gene of pAX01.
P52	GanB-Cnf	CAATGGCAGCGGCATATCC	Confirmation of chromosomal integration at *Bs lacA* locus. Primers bind to *ganB* downstream of *lacA* (*ganA*), and to the *xylR* gene of pAX01.
P53	XylR-Cnf	GGAGCGGTTTCTATCGTTATTGATTC	
P54	Ycg-Cnf	GGAAGCGTTCACAGTTTCG	Confirmation of chromosomal integration at *Bs amyE* locus. Primers bind to *ycgB* upstream of *amyE*, and to the *lacZ* gene of pBS1C*lacZ*.
P55	LacZ-Cnf	TCCTGGAGCCCGTCAGTATC	
P56	Ldh-Cnf	CAATGACCACAAGCTCATCTG	Confirmation of chromosomal integration at *Bs amyE* locus. Primers bind to *ldh* downstream of *amyE*, and to the *cat* gene of pBS1C*lacZ*.
P57	Cat-Cnf	CTATTCAGGAATTGTCAGATAGGC	

Restriction site sequences are underlined. The mutations introduced in the -35 and -10 promoter regions of the σ^I-dependent promoter of *C. thermocellum rgl11* are indicated in red and underlined. *Bc*, *Bacteroides cellulosolvens*; *Bs*, *Bacillus subtilis*; *Ct*, *Clostridium thermocellum*.

[a] Primer designed for cloning with the In-Fusion HD Cloning Kit.

[b] X represents undisclosed base in the proprietary SMARTer oligo sequence.

Table S3. Plasmids constructed in the present work.

#	Plasmid use and name	Relevant genotype[a]	Brief description
	Analysis of alternative σ^{I} factor in *B. subtilis*		
1	pAX01[b]	*bla lacA3' xylR* P_{xylA} *erm lacA5'*	Integration vector
2	pAX01-Bc-SigI11	pAX01 P_{xylA}-*sigI11*$_{Bc}$	pAX01-derived plasmid for the expression of *B. cellulosolvens* σ^{I11} or *C. thermocellum* σ^{I3}
3	pAX01-SigI3[b]	pAX01 P_{xylA}-*sigI3*$_{Ct}$	
4	pBS1C-LacZ[b]	*bla amyE5' cat* mcs *rfp* mcs *lacZ amyE3'*	Integration vector with a LacZ reporter gene
5	pProm-Ct-Cel48S	pBS1CLacZ P_{cel48S_Ct}-*lacZ*	pBS1C-LacZ-derived plasmids which have the σ^{I}-dependent promoter of *C. thermocellum cel48S*
	Construction of the GFP-LacZ reporter system		
6	pBS1C-GFP-LacZ[b]	*bla amyE5' cat* mcs *rfp* mcs *gfp-lacZ amyE3'*	Integration vector with a GFP-LacZ reporter operon
7	pProm-Ct-Rgl11A	pBS1C-GFP-LacZ P_{rgl11A_Ct}-*gfp-lacZ*	pBS1C-GFP-LacZ-derived plasmids which have wild type and mutant version of the σ^{I3}-dependent promoter of *C. thermocellum rgl11A*
8	pProm-Ct-Rgl11A-Mut1	pBS1C-GFP-LacZ $P_{rgl11A\_Ct\text{-}Mut1}$-*gfp-lacZ*	
9	pProm-Ct-Rgl11A-Mut2	pBS1C-GFP-LacZ $P_{rgl11A\_Ct\text{-}Mut2}$-*gfp-lacZ*	
10	pProm-Ct-Rgl11A-Mut3	pBS1C-GFP-LacZ $P_{rgl11A\_Ct\text{-}Mut3}$-*gfp-lacZ*	
11	pProm-Ct-Rgl11A-Mut4	pBS1C-GFP-LacZ $P_{rgl11A\_Ct\text{-}Mut4}$-*gfp-lacZ*	
12	pProm-Ct-Rgl11A-Mut5	pBS1C-GFP-LacZ $P_{rgl11A\_Ct\text{-}Mut5}$-*gfp-lacZ*	
13	pProm-Ct-Rgl11A-Mut6	pBS1C-GFP-LacZ $P_{rgl11A\_Ct\text{-}Mut6}$-*gfp-lacZ*	
14	pProm-Ct-Rgl11A-Mut7	pBS1C-GFP-LacZ $P_{rgl11A\_Ct\text{-}Mut7}$-*gfp-lacZ*	
15	pProm-Ct-Rgl11A-Mut8	pBS1C-GFP-LacZ $P_{rgl11A\_Ct\text{-}Mut8}$-*gfp-lacZ*	
16	pProm-Ct-Rgl11A-Mut9	pBS1C-GFP-LacZ $P_{rgl11A\_Ct\text{-}Mut9}$-*gfp-lacZ*	
17	pProm-Ct-Rgl11A-Mut10	pBS1C-GFP-LacZ $P_{rgl11A\_Ct\text{-}Mut10}$-*gfp-lacZ*	
18	pProm-Ct-Rgl11A-Mut11	pBS1C-GFP-LacZ $P_{rgl11A\_Ct\text{-}Mut11}$-*gfp-lacZ*	
19	pProm-Ct-Rgl11A-Mut12	pBS1C-GFP-LacZ $P_{rgl11A\_Ct\text{-}Mut12}$-*gfp-lacZ*	
20	pProm-Ct-Rgl11A-Mut13	pBS1C-GFP-LacZ $P_{rgl11A\_Ct\text{-}Mut13}$-*gfp-lacZ*	
21	pProm-Ct-Rgl11A-Mut14	pBS1C-GFP-LacZ $P_{rgl11A\_Ct\text{-}Mut14}$-*gfp-lacZ*	
22	pProm-Ct-Rgl11A-Mut15	pBS1C-GFP-LacZ $P_{rgl11A\_Ct\text{-}Mut15}$-*gfp-lacZ*	
23	pProm-Ct-Rgl11A-Mut16	pBS1C-GFP-LacZ $P_{rgl11A\_Ct\text{-}Mut16}$-*gfp-lacZ*	
24	pProm-Ct-Rgl11A-Mut17	pBS1C-GFP-LacZ $P_{rgl11A\_Ct\text{-}Mut17}$-*gfp-lacZ*	
25	pProm-Ct-Rgl11A-Mut18	pBS1C-GFP-LacZ $P_{rgl11A\_Ct\text{-}Mut18}$-*gfp-lacZ*	
26	pProm-Bccel_3806	pBS1C-GFP-LacZ P_{Bccel_3806}-*gfp-lacZ*	pBS1C-GFP-LacZ-derived plasmids which have *B. cellulosolvens* σ^{I11}-dependent promoters
27	pProm-Bccel_5179	pBS1C-GFP-LacZ P_{Bccel_5179}-*gfp-lacZ*	
28	pProm-Bccel_5619	pBS1C-GFP-LacZ P_{Bccel_5619}-*gfp-lacZ*	
29	pProm-Bccel_5622	pBS1C-GFP-LacZ P_{Bccel_5622}-*gfp-lacZ*	
30	pProm-Bccel_5627	pBS1C-GFP-LacZ P_{Bccel_5627}-*gfp-lacZ*	
31	pProm-Bccel_5541	pBS1C-GFP-LacZ P_{Bccel_5541}-*gfp-lacZ*	

[a] *Ct*, *C. thermocellum*; *Bc*, *B. cellulosolvens*; mcs, multiple cloning sites; *rfp*, red fluorescence protein gene; *gfp*, green fluorescence protein gene.
[b] pAX01 was obtained from the Bacillus Genetic Stock Center. pAX01-SigI3, pBS1CLacZ and pBS1C-GFP-LacZ were constructed in previous works[1–3]. pET-28a(+) was obtained from Novagen.

Table S4. Alignment of predicted promoters upstream of the *B. cellulosolvens* σ^{I}-RsgI gene operons.

Locus tag[a]	Gene product	RsgI-sensory-element[b]	Promoter sequence (5'→3')	5'-UTR[c]
Bccel_0204-Bccel_0205	σ^{I1}-RsgI1	PA14-PA14	aa*cgacct***AAAA**att-atttcatt**CGAA**tattta	240
Bccel_0630-Bccel_0629	σ^{I2}-RsgI2	FN3	tg*ctctta***AAAA**tcatacttcttat**CGAA**caatat	15
Bccel_0725-Bccel_0726	σ^{I3}-RsgI3	CBM3	ct*atactg***AAAA**atttgaattttat**CGAA**atatat	16
Bccel_2224-Bccel_2225	σ^{I4}-RsgI4	UNK	gg*aactat***AAAA**tct-ac-tcttct**CGT**cactgct	541
Bccel_2755-Bccel_2756	σ^{I5}-RsgI5	UNK	Not identified	
Bccel_3092-Bccel_3091	σ^{I6}-RsgI6	CBM42	cc*gaactc***AAAA**ataaattcgatta**CGAA**ttaatc	184
Bccel_3399-Bccel_3398	σ^{I7}-RsgI7	UNK	Not identified	
Bccel_5133-Bccel_5134	σ^{I8}-RsgI8	UNK	gc*cacata***AAAA**tattttatactta**CG**cctttatt	14
Bccel_5156-Bccel_5155	σ^{I9}-RsgI9	UNK	ac*cgaaca***AAAA**caagcaaaaattt**CGAA**tatact	19
Bccel_5614-Bccel_5615	σ^{I10}-RsgI10	UNK	Not identified	
Bccel_5622-Bccel_5623	σ^{I11}-RsgI11	PA14-CBM35	at*atcccg***AAAA**agttttaaatgca**CG**c**A**tatata	15
Bccel_5637-Bccel_5636	σ^{I12}-RsgI12	CBM3	gc*cacatc***AAAA**atttactttactc**CGAA**acacta	22
Bccel_0208	σI-RsgI fused	UNK	Not identified	
Bccel_0210	σ^{I}-RsgI fused	UNK	Not identified	
Bccel_2219	σ^{I}-RsgI fused	UNK	Not identified	
Bccel_4550	σ^{I}-RsgI fused	UNK	ct*tgactc***AAAA**tgtacagctaaac**CG**ctcgctat	72
General motifs of σ^{I}-dependent promoters			········**AAAA**··(12-14)N···**CGAA**	

Nucleotides that match the general motifs of σ^{I}-dependent promoters are shown in bold capital fonts. The region of specificity is italicized.

[a] *B. celullosolvens* DNA sequences were obtained from GeneBank (LGTC01000001.1).

[b] FN3, fibronectin type 3 domain; CBM, carbohydrate binding module; UNK, unknown.

[c] Distances between the promoter region sequences used for the alignment and the first codon of corresponding genes are shown in the column labeled 5'-UTR (5'-untranslated region).

Table S5. Predicted σ^I-dependent promoters of *Bacteroides cellulosolvens*.

Locus tag[a]	Gene product	Promoter sequence 5'→3'	5' UTR
Bccel_0022	GH10	GAAGAAAGAAATATCAGAGTAGATATCGTCGGCTGG	31
Bccel_0051-0050	GH57	GTCTTATTAAAATAATA-CTAAAATTCGATTAGCTT	57
Bccel_0147	CBM25	TCCGCTATAAATTTTAC-TCATGTGTCGACTTATCT	123
Bccel_0290	GH9-CBM3-DocII	GTGTGAAAAAATTTTGT-GTATTACACGCCATAATT	241
Bccel_0386	Cellulase-DocII	TATCCGGGAAAACAA-A-A-GAGAACCGTTCAATAA	292
Bccel_0442	Esterase	GTCATGATAAATTGT-G-GTGCGTAACGCACCACAA	128
Bccel_0442	Esterase	AGCTACAAAAATTCAAACGGGACTACCGTTGTTACT	427
Bccel_0446	GH8-DocII-Polysaccharide deacetylase	ATCGACATAAATATA-T-TCTTTTTACGTATTTTGA	57
Bccel_0465	GH8-DocII-XynB_like	AATCGTCAAAAAATTTT-TCTGTTTACGAATATTAC	928
Bccel_0465	GH8-DocII-XynB_like	TTATAGTTAAAAAATAACATAAAAATCGTCAAAAAA	950
Bccel_0468	GH81-DocII	TTCTACTGAAATTAGTT-ATTAACACCGCTATTCTA	289
Bccel_0485	GH9-CBM3-CBM3-Doc	GTACACTGAAATTATAT-GAAATGTACGAATTTTAT	84
Bccel_0485	GH9-CBM3-CBM3-Doc	CTACAAGTAAACTAATA-CCTTCAACCGATAACTCA	347
Bccel_0485	GH9-CBM3-CBM3-Doc	TACGGGATAAACAACGT-GTACTATCCGATACACTT	413
Bccel_0485	GH9-CBM3-CBM3-Doc	ATTCCTGTAAAATAA-A-AAACTCTTCGTCCTTGAA	506
Bccel_0497-0496	GH53-DocII	TACCCATAAAATCGCAG-TTTCTTTTCGCATTAGTA	57
Bccel_0519	GH9-CBM3-CarboxypepD_reg-DocII	TTAATGATAAAAGCA-G-TAAAATGCCGTAATACGA	169
Bccel_0527	DocII-XynE_like	TCCGACATAAAAATT-A-TTATCCAACGAAATATTT	173
Bccel_0535	DokII-E_set-CotH	ACAGTTACAAAAATA-A-ACAATTTGCGACTAAAGT	53
Bccel_0535	DokII-E_set-CotH	TCATTAATAAAACCT-C-CCTATAAACGTTTTTTTT	427
Bccel_0571	UNK-CarboxypepD_reg-DocII	AAACTAACAAACAAT-T-GAATTAATCGTATATAAA	134
Bccel_0665	CohI-PA14-DUF11-3xSLH	ATTATGCCAAAAAAGAG-CATTGAAACGACAACCGA	333
Bccel_0690	GH8-DocII-Polysaccharide dacetylase	AAAAATATAAAAACA-C-A-ATATTTCGAAAAAAAA	418
Bccel_0690	GH8-DocII-Polysaccharide dacetylase	TCATATCTAAATAATATGGTCATATCCGTTGAATCA	503
Bccel_0723	Cellulase_GH5-DocII	CGAACTGTAAAGTTATA-TATCAGTTCGATAACCAT	67
Bccel_0723	Cellulase_GH5-DocII	CCCTACTCAAATAAGTT-TGTTTTTTCGAATTATTA	112
Bccel_0839	DocI-DocI	TATGTAACAAAAGGT-T-ATTACGAACGATATATTT	103
Bccel_0849	2xSLH-UNK	TCCTTATGAAAAAAACT-TTTTATGTCGAATAATTT	71
Bccel_0881	GH9	CAAACTTTAAAGTTATA-GATAAGATCGATATATAA	120
Bccel_0895	GH48-DocII	TAATTAACAAATGTTGTTATAGATGCCGAAAAGTAA	112
Bccel_0922	CotH-CBM_4_9-DocI	TTGTAGTAAAACGCCCG-GGCAAAATCGTTAGGCAT	125
Bccel_0922	CotH-CBM_4_9-DocI	ATGTATTTAAATGTTATATTTAGAAGCGTAAAGTTC	160
Bccel_1025	CohII-3xSLH	TTTTTTATAAAGACATTATGAATAAACGACGGAGCT	355
Bccel_1216	Cellulase_GH5-F5/8_C-CarboxypepD-DocII	ACCCACATAAATTTAAA-AAAACATACGAATTATTA	74
Bccel_1250	CBM35-GH26_Mannase-CarboxypepD-DocII	TTATAGTAAAATTGTTTATATAGAATCGATTATAGA	84
Bccel_1253	12xRCC1-CarboxypepD_reg-DocII	TCCTCCTTAAAAGTAGA-ATTAAAAGCGATAATTTT	177
Bccel_1260	GH11-DocII	ATGTAAATAAATGGGCAGAGAGTGATCGAATGATTA	272
Bccel_1262	XynB_like-DocII	ACCAGCTTAAAAATTCA-ATTGATTTCGAATTTATA	56
Bccel_1425	GH10	CACATTAAAAATATGAA-AAATATGACGTATAATAA	196
Bccel_1485	GH9-CBM3-CarboxypepD_reg-DocII	TTTAATTGAAATAGATCAATTAAAAGCGTTTAACTT	84
Bccel_1485	GH9-CBM3-CarboxypepD_reg-DocII	TCCAATGTAAAGAAT-T-GCCCCCAACGCTCCATAA	128
Bccel_1485	GH9-CBM3-CarboxypepD_reg-DocII	AAGTTAGCAAAATTC-A-T-TTTATCCGAATAAAAA	175
Bccel_1485	GH9-CBM3-CarboxypepD_reg-DocII	TATTAGATAAACTCCAATCTTTCTATCGTAATATTC	633
Bccel_1712	GH43	AAAAATGTAAAGCAA-A-CGGTTAATCGATGCAATG	131
Bccel_1738	GH43_RICIN-DocII-GH16	TTACAGATAAAGTTATA-TATCAGTTCGCTTGGAAT	126
Bccel_1743	GH30-DocII	TAGTACTCAAAAAGT-A-ATATCATACGAAATTTAT	111
Bccel_1767	SasC_Mrp_aggreg-SLH-SLH-SLH	GAAAACTCAAAAAGT-T-GGAGATTTCGAAATAATA	201
Bccel_1767	SasC_Mrp_aggreg-SLH-SLH-SLH	GTTACTTCAAAATAC-A-G-CTATGACGATGGTAAA	285
Bccel_1798	UNK-DocII-UNK	ACTGATCCAAATTTC-C-AGTTCTATCGTTATCTTC	339
Bccel_1815	GH44-CohI-DocI	ATTATATTAAAAGTATT-ATTTGCCCCGCCAAAGGT	495
Bccel_1985	PKD-5xFN3-PKD-29xFN3-3xCARB-PKD-CARB-etc…	CATACAAGAAATTAATA-AATATAGTCGTAAAAAGA	127

Bccel_1985	PKD-5xFN3-PKD-29xFN3-3xCARB-PKD-CARB-etc…	CAATTGTGAAATGGT-C-C-GTATTTCGATCATTTT	393
Bccel_2050	5xCohII-CBM3-6xCohII-DocI	TTAACATTAAAAAGAAT-AAGTACAACGTTTGACAT	1221
Bccel_2050	5xCohII-CBM3-6xCohII-DocI	TATTCGCTAAATTTG-C-TTCATGGTCGCCTTGGTT	489
Bccel_2186	11xRCC1-DocII	CCGAACTGAAATTTCAC-CCAATCAACGAACTTTTC	298
Bccel_2277	DocI-CohII-CohII-CohII	TTAGGTATAAAAATA-G-A-CAAAACCGTTCTAGTA	151
Bccel_2479	CarboxypepD_reg-DocI-UNK-SERPIN	CCCAAATCAAATAAA-A-A-ACATGTCGAATTTAGA	276
Bccel_2492	Cellulase_GH5-UNK-Cellulase_GH5-DocII	TTTTTTTGAAAAGAA-A-T-TTGCAGCGAATAATGA	457
Bccel_2557	CBM_4_9-E_set-GH9-DocII	ATGTTTGTAAAAATT-T-ATTGCAGTCGTCTGTAAA	506
Bccel_2757	Copper amine oxidase	CACCCCAAAAATTTATATACACTTTCCGAATGTATA	39
Bccel_2898	RCC1-UNK-RCC1- Copper amine oxidase	CCCTCCGGAAACATTTT-ATCATAAGCGTATAAAAA	457
Bccel_2918	CohI-EVI2A-3xSLH	TTATTGTAAAAGGCAAC-AGAATTTCCGTATATATA	116
Bccel_2922	CohI-Copper amine oxidase-like domain-containing protein	ATAGCCGAAAAAGGGCT-ATTCCGTGCGAACTGATA	111
Bccel_3097	GH43-CBM6-DocII	CCTAACTTAAAAAAGTT-CAACAATACGAAAATGTA	283
Bccel_3114	XynB2-CBM6-DocII	TTGCAAATAAACCGA-T-TAAGGTACCGCAAGCTTC	37
Bccel_3120	UNK	ATACAGTCAAAATAACA-TATAACCACGTTCAATCC	21
Bccel_3121	UNK	CCTGACTTAAATAAATA-TATTACTGCGAATAAGTA	65
Bccel_3125	GH27-CBM6-DocII	CCTAATTTAAACAATTA-TATTACTGCGAATAAGTA	82
Bccel_3128	DocII-DUF2233	CTCCGCTTAAATTTATT-TTTTGTTACGTATATATT	235
Bccel_3158	CBM25	ATTTTATCAAATTGC-A-TCCATTATCGTCCATTAT	102
Bccel_3158	CBM25	AATAAATAAAACGGAATAATTTTCTCCGACTTTCAA	196
Bccel_3366	FN3-vWFA-SH3	CGATTTAAAAAAATTTG-AAGATTATCGAAGAACAA	94
Bccel_3440	CBM3 (peptide)	CTTGATGGAAATCCT-A-T-AACGCCCGAATCTTTA	216
Bccel_3452	CohII-Doc-7xVCBS	AAAGTTTGAAAAATG-G-TAGATTTACGATTTGTAT	46
Bccel_3452	CohII-Doc-7xVCBS	TATATCTTAAAAACT-A-A-CTTTTTCGTAGAAAAT	197
Bccel_3452	CohII-Doc-7xVCBS	ACCTGTCTAAACACC-A-G-AGCAAGCGAAAATGCA	719
Bccel_3459	14xRCC1-DocII	CAAAAAGAAAAATATAC-ATAAACTCCGCCTAAGAT	327
Bccel_3527	GH8-DocII	TATTAATTAAAAAAGCA-ATAATTAGCGAATGTATC	519
Bccel_3562	UNK-CarboxypepD_reg-DocII-Inhibitor_I42-SERPIN	AAAGTTACAAACATGTT-TTAATTTACGAATTAAGG	10
Bccel_3600	UNK	CCCTCCTTAAATTTA-A-AAAACCTCCGAACAATAT	74
Bccel_3672	7xCohI	TTTTTTCAAAAATGCGA-ACGAAAACCGTAAGTTTA	258
Bccel_3672	7xCohI	TAAACTAGAAAAGTGCT-TAAAGTTTCGTTGCTATA	36
Bccel_3733	GH11-PT-GH10-DocII-CE4	TTATAGTTAAACAAAAG-TTCAAAAGCGAAAGGAGA	7
Bccel_3749	2xCBM_4_9-DUF303-CE4-3xCBM_4_9-GH10-2xCBM9-2xSLH	TGATTACTAAAGATATT-TATTACTGCGTATTTATG	373
Bccel_3749	2xCBM_4_9-DUF303-CE4-3xCBM_4_9-GH10-2xCBM9-2xSLH	AGCTTTAAAAAGCTATG-CATATGTTCGTTTTATTA	248
Bccel_3806	Pectate_lyase	ACCACCCTAAAATTTTA-AGTATGTACGAATTAATT	63
Bccel_3814	GH10-CBM6-DocII	AAATTACCAAACCTT-G-ATTGTTGTCGTAAGAAAA	318
Bccel_3832	DocII-Beta_propel	GTGTGTCAAAATAATAT-AATAATAACGTACAAAAT	223
Bccel_3834	CBM_4_9-E_set-GH9-DocII	TATTTAACAAATATA-T-G-TTTAAGCGAATTGACA	277
Bccel_3834	CBM_4_9-E_set-GH9-DocII	TACGCCTAAAATAAAATGACAGATCACGTATATAGT	781
Bccel_3923	E_set-Alpha amylase	CATGATCAAAAAATAAC-TTCCGCTACGTTTTTTCA	198
Bccel_3943	Cellulase_GH5-CarboxypepD_reg-DocII	TATTCAATAAAATAT-A-AGTATACCCGTATTTATA	100
Bccel_4009	GH3-CarboxypepD_reg-DocII	AAGGCACCAAAGCAACA-TTTGCTTTCGTTAAGTTA	102
Bccel_4138	UNK	TCTTATCTAAAATTTAG-ATATCAAACGAATCAAAA	561
Bccel_4153	UNK	GGCACAAAAAAATTTAT-ATAACTTTCGAACTACTA	87
Bccel_4177	GH30-CarboxypepD_reg-DocII	GAATTATTAAATTAA-G-GAATACTTCGCTTAAAAA	118
Bccel_4177	GH30-CarboxypepD_reg-DocII	TAGCACTTAAAATAA-T-ATTTTCTGCGAATTATTA	143
Bccel_4177	GH30-CarboxypepD_reg-DocII	TATTATATAAAAAGTTCAATAGAACACGCTTCACTG	325
Bccel_4379	GH43	AACTTAATAAAATATTA-TTAGCACTCGATGGCGTT	190
Bccel_4452	DocII-4xLRR	GATTTTGTAAATCTTGC-TCATTTTCCGCAGAAGTT	359
Bccel_4497	GH127-DocII	TATTGATGAAAGAAA-A-ATTACTTACGCTTTTATT	343
Bccel_4501	CarboxypepD_reg-DocII-GH16-2xCBM_4_9-CBM6-2xCBM_4_9	CGAATTTTAAAATGGGT-TAATAAAACGATAATTCT	438
Bccel_4501	CarboxypepD_reg-DocII-GH16-2xCBM_4_9-CBM6-2xCBM_4_9	TGAAACACAAAATTCGG-GACACTTCCGTTTTACAT	888

Bccel_4501	CarboxypepD_reg-DocII-GH16-2xCBM_4_9-CBM6-2xCBM_4_9	GGCGACGTAAACAAA-T-GCTTGACTCGCTGCCCTA	1040
Bccel_4501	CarboxypepD_reg-DocII-GH16-2xCBM_4_9-CBM6-2xCBM_4_9	GCAATGCAAAAATTGAATATAATGAGCGACAAATGC	1095
Bccel_4616	CE4	TAATCAATAAAAATATA-TTGAATAACGATAAGAAT	131
Bccel_4619	2xWG_bet_rep-UNK-DUF4163-DUF3298-UNK	GAATTGCGAAAGTCATT-GTAGATATCGTACTTTGA	49
Bccel_4619	2xWG_bet_rep-UNK-DUF4163-DUF3298-UNK	AATAGAATAAAAGAA-G-A-GAATTGCGAAAGTCAT	68
Bccel_4653	UNK-DocII-UNK	TCGCACATAAATTACAG-CAAAAATTCGTAGCAATT	50
Bccel_4684	CBM3-2xCBM6-PT-F5_F8_type_C-UNK-vWFA-UNK	ATAATATCAAAGAGA-T-T-GTTTTTCGATTAATTT	223
Bccel_4693	CBM3-4xCohII	TGAAATACAAATGCTCT-TTTCCAAGCGTTATATAA	195
Bccel_4762	HP	CGTACACAAAAACCATA-TTGACAGACGAACAAACG	100
Bccel_4836	UNK-Disaggr_repeat-CarboxypepD_reg-DocII	TCCCTATTAAAAAAATT-GGCAACTTCGAACAATAT	29
Bccel_4965	6xFN3-SLH	CCATAATCAAATAGGCA-CAAAAAGCCGAAATAATT	27
Bccel_5179	Pectinesterase-CBM35-DocII	CATGCCCTAAAATAAAT-TGATTCTACGCATTGTTT	85
Bccel_5193	DocII-Esterase	GTGGACATAAAAATACG-GTTATTTTCGAATTCATT	48
Bccel_5216	CBM_4_9-E_set-GH9-DocII	CATATTGTAAAGCAT-A-ATAATGTTCGATATAAATA	94
Bccel_5269	LTD-Carboxypeptidase-DocII-DUF4353	AAAACAGTAAACAAA-G-T-CGCTTGCGATTTTGCA	115
Bccel_5275	CarboxypepD_reg-DocII-UNK	CATTATAGAAATGGGGCGTTGTACTACGATTATAAA	254
Bccel_5319	Amidohydrolase	TTGTACACAAAATAA-T-TTTTTGTACGAATATTAC	461
Bccel_5319	Amidohydrolase	ATTTTAATAAATGTA-T-CATATAATCGAAAGTAAT	17
Bccel_5354	2xFN3-UNK	AAACCGCAAAAAAAATG-TTGGATTCCGAAAAAATG	90
Bccel_5375	LRR-Copper amine oxidase-DUF5050	CTATTTATAAAATAA-A-T-ATTGACCGATATAACC	161
Bccel_5400	CohII-DocII	TGCCCTCTAAATAATAAAAGATTAGCCGTTTTCCAT	652
Bccel_5400	CohII-DocII	TGTTATTAAAATTCAATATAAGAGGGCGATAAAATA	418
Bccel_5508	Cellulase_GH5-DocII	TTTATATGAAAGTAATTTATGGATACCGAAATGTAA	56
Bccel_5509	UNK-DocII	ATATATAAAAAAATGTG-TAATACACCGTTTACATC	128
Bccel_5538	GH20-GH115-UNK-CBM6	ATTATAGTAAATTTG-T-TTCAAACACGAATGTTAT	224
Bccel_5541	GH30-DocII	TACCCCACAAAAAGGCT-TGTTGATTCGCATTATTT	41
Bccel_5619	RGAE-PT-DocII	GTACCCTAAAAATAA-T-ATTCTTTTCGCATATTAA	92
Bccel_5625	E_set-CotH-DocII	TACTAGCAAAAATATAT-AAATCAGTCGCAGTTTAA	290
Bccel_5627	RGL11-DocII	TATCCCAAAAAACATAA-AAAATTTACGTATTTTTA	89
Bccel_5646	UNK-2xSLH-UNK	CATGATCGAAAAATA-A-TAGCAAAGCGAAATTTCC	12
Bccel_5682	Copper amine oxidase	TAAAAGTGAAAAAAA-C-CGAATATTCGACAAAAAA	78
Bccel_5725	Esterase-DocII	CAGAACACAAAAAAATA-TCTTTTAACGAACAATAT	201
Bccel_5725	Esterase-DocII	ATATTATGAAATGCTAT-AAAATTTGCGAACAGCAA	168
Bccel_5726	CBM9-Esterase-3xSLH	TTTTAATGAAAATAAGC-AATTTTTTCGTTGAATTA	193
Bccel_5883	Copper amine oxidase-like domain-containing protein	GCCCAGATAAAGTAT-A-TGGATAAGCGTTCAACCA	388
Bccel_5883	Copper amine oxidase-like domain-containing protein	CAAGGGAAAAATTATGAATTCAAAGCCGATTTATAG	560
Bccel_5883	Copper amine oxidase-like domain-containing protein	TATAACATAAAGACT-T-TTCAAGTTCGCAATATAA	96
General motifs of σ[I]-dependent promoters[b]		········AAA····(12-15)N···CGAA	

[a] *B. celullosolvens* DNA sequences were obtained from GeneBank (LGTC01000001.1).

[b] The general motifs of σ[I]-dependent promoters are shown in Figure 1A.

CARB, Cell adhesion related domain found in bacteria; CarboxypepD_reg, carboxypeptidase regulatory-like domain; CBM, carbohydrate binding module; CE, carbohydrate esterase; CotH, spore coat protein H; Disaggr_repeat, disaggregatase related repeat; Doc, dockerin; DUF, Domain of unknown function; EVI2A, Ectropic viral integration site 2A protein; E_set, "early" set domains of sugar utilizing enzymes; FN3, Fibronectin type 3 domain; F5/8_C, coagulation factor 5/8 type domain; GH, glycoside hydrolase; ; LRR, leucine rich repeats; LTD, lamin tail domain; PA14, anthrax protective antigen 14 domain; PKD, polycystic kidney disease domain; PT, PT repeat; RCC1, Regulator of chromosome condensation repeat; RICIN, Ricin-type beta-trefoil lectin domain-like; RGAE, Ramnogalacturan_acetylesterase; RGL, Rhamnogalacturonan lyase; SasC_Mrp_aggreg, intercellular aggregation domain; SERPIN, serine proteinase inhibitors; SH3, src homology-3 domain; SLH, S-layer homology domain; UNK, unkown; VCBS, Repeat domain in Vibrio, Colwellia, Bradyrhizobium and Shewanella; XynB_like, similar to *Ruminococcus flavefaciens* XynB (Endo-1,4-beta-

xylanase); vWFA, von Willebrand factor type A; WG_bet_rep, WG containing repeats; XynE_like, similar to the putative arylesterase/acylhydrolase from *Prevotella bryantii* XynE.

Table S6. ECF σ factors of *Bacteroides cellulosolvens*.

**Locus tag[a]**	**ECF group[b]**	**Locus tag[a]**	**ECF group[b]**
Bccel_0159	ECF01	Bccel_4188	ECF01
Bccel_0476	ECF01	Bccel_4398	ECF01
Bccel_0498	UNK	Bccel_4525	ECF01
Bccel_0837	ECF01	Bccel_4677	UNK
Bccel_1048	ECF30	Bccel_4936	ECF01
Bccel_1264	UNK	Bccel_5104	UNK
Bccel_1521	ECF01	Bccel_5167	UNK
Bccel_3425	UNK	Bccel_5212	UNK
Bccel_3430	UNK	Bccel_5376	UNK
Bccel_4023	ECF01	Bccel_5478	UNK
Bccel_4049	ECF01	Bccel_5543	ECF01

[a] *B. celullosolvens* DNA sequences were obtained from GeneBank (LGTC01000001.1).
[b] The identification of ECF groups was performed with the program ECFfinder (http://ecf.g2l.bio.uni-goettingen.de:8080/ECFfinder/start.html).

References

1. Muñoz-Gutiérrez, I. *et al.* Decoding biomass-sensing regulons of Clostridium thermocellum alternative Sigma-I factors in a heterologous Bacillus subtilis host system. *PLoS One* **11,** e0146316 (2016).

2. Radeck, J. *et al.* The Bacillus BioBrick Box: generation and evaluation of essential genetic building blocks for standardized work with Bacillus subtilis. *J. Biol. Eng.* **7,** 29 (2013).

3. Ortiz de Ora, L. *et al.* Revisiting the regulation of the primary scaffoldin gene in Clostridium thermocellum. *Appl. Environ. Microbiol.* **83,** e03088-16 (2017).
